# The role of endometrial B cells in normal endometrium and benign female reproductive pathologies: a systematic review

**DOI:** 10.1093/hropen/hoab043

**Published:** 2021-12-25

**Authors:** Mengni Shen, Elizabeth O’Donnell, Gabriela Leon, Ana Kisovar, Pedro Melo, Krina Zondervan, Ingrid Granne, Jennifer Southcombe

**Affiliations:** 1 Nuffield Department of Women’s and Reproductive Health, University of Oxford, Oxford, UK; 2 Tommy’s National Centre for Miscarriage Research, Institute of Metabolism and Systems Research, University of Birmingham, Birmingham, UK

**Keywords:** endometrium, immunology, endometriosis, female infertility, recurrent miscarriage, infertility, B cells

## Abstract

**STUDY QUESTION:**

What are the similarities and differences in endometrial B cells in the normal human endometrium and benign reproductive pathologies?

**SUMMARY ANSWER:**

Endometrial B cells typically constitute <5% of total endometrial CD45^+^ lymphocytes, and no more than 2% of total cells in the normal endometrium, and while their relative abundance and phenotypes vary in benign gynaecological conditions, current evidence is inconsistent.

**WHAT IS KNOWN ALREADY:**

B cells are vitally important in the mucosal immune environment and have been extensively characterized in secondary lymphoid organs and tertiary lymphoid structures (TLSs), with the associated microenvironment germinal centre. However, in the endometrium, B cells are largely overlooked, despite the crucial link between autoimmunity and reproductive pathologies and the fact that B cells are present in normal endometrium and benign female reproductive pathologies, scattered or in the form of lymphoid aggregates (LAs). A comprehensive summary of current data investigating B cells will facilitate our understanding of endometrial B cells in the endometrial mucosal immune environment.

**STUDY DESIGN, SIZE, DURATION:**

This systematic review retrieved relevant studies from four databases (MEDLINE, EMBASE, Web of Science Core Collection and CINAHL) from database inception until November 2021.

**PARTICIPANTS/MATERIALS, SETTING, METHODS:**

The search strategy combined the use of subject headings and relevant text words related to ‘endometrium’, ‘B cells’ and B-cell derivatives, such as ‘antibody’ and ‘immunoglobulin’. Non-benign diseases were excluded using cancer-related free-text terms, and searches were limited to the English language and human subjects. Only peer-reviewed research papers were included. Each paper was graded as ‘Good’, ‘Fair’ or ‘Poor’ quality based on the NEWCASTLE-OTTAWA quality assessment scale. Only ‘Good’ quality papers were included.

**MAIN RESULTS AND THE ROLE OF CHANCE:**

Twenty-seven studies met the selection criteria and were included in this review: 10 cross-sectional studies investigated B cells in the normal endometrium; and 17 case–control studies compared the characteristics of endometrial B cells in control and benign female reproductive pathologies including endometritis, endometriosis, infertility, abnormal uterine bleeding, endometrial polyps and uterine fibroids. In all studies, B cells were present in the endometrium, scattered or in the form of LAs. CD20^+^ B cells were more abundant in patients with endometritis, but the data were inconsistent as to whether B-cell numbers were increased in endometriosis and patients with reproductive pathologies.

**LIMITATIONS, REASONS FOR CAUTION:**

Although only ‘good’ quality papers were included in this systematic review, there were variations in patients’ age, diagnostic criteria for different diseases and sample collection time among included studies. Additionally, a large number of the included studies only used immunohistochemistry as the identification method for endometrial B cells, which may fail to provide an accurate representation of the numbers of endometrial B cells.

**WIDER IMPLICATIONS OF THE FINDINGS:**

Histological studies found that endometrial B cells are either scattered or surrounded by T cells in LAs: the latter structure seems to be under hormonal control throughout the menstrual cycle and resembles TLSs that have been observed in other tissues. Further characterization of endometrial B cells and LAs could offer insights to endometrial B-cell function, particularly in the context of autoimmune-associated pathologies, such as endometriosis. Additionally, clinicians should be aware of the limited value of diagnosing plasma cell infiltration using only CD138.

**STUDY FUNDING/COMPETING INTEREST(S):**

This study was funded by Finox Biotech. The authors have no conflicts of interest to declare.

**PROSPERO REGISTRATION NUMBER:**

This systematic review was registered in PROSPERO in January 2020 (PROSPERO ID: CRD42020152915).

WHAT DOES THIS MEAN FOR PATIENTS?

The endometrium is the lining of the uterus. Immune cells in the endometrium are important to prevent infection and also help with the establishment of pregnancy. This study investigates a type of immune cell that typically makes antibodies, but can have other immune functions, called B cells. B cells are a minor cell population in the endometrium, which have long been perceived as insignificant. However, recent studies have shown endometrial B cells have distinct characteristics when compared with circulating B cells and may be actively involved in shaping the endometrial immune environment, highlighting the importance of understanding the role of B cells in the endometrium.

Through studying the published papers that investigate endometrial B cells, we have identified those that provide good quality research that contributes to our knowledge of the cells in health and non-cancerous diseases, such as endometriosis, infertility, recurrent implantation failure, recurrent pregnancy loss and endometritis. Endometrial B cells are reported to be significantly increased in patients with endometritis compared with healthy women; however, B-cell numbers are inconclusive in other gynaecological diseases. Additionally, B cells are often observed in the centre of immune cell aggregates, and it is possible that these immune cell aggregates are an indication of active B-cell development in the endometrium, therefore, future studies are needed to explore their cellular interactions and functions.

## Introduction

B cells, often identified by pan B-cell markers CD19, CD20 or CD22, are a part of the adaptive immune system. Apart from their well-known antibody production capacities, B cells also act as professional antigen presenting cells. After their initial development in the bone marrow, immature B cells migrate to the secondary lymphoid organs (SLOs), such as lymph nodes, tonsils, spleen or mucosal-associated lymphoid tissue (MALT), where B-cell activation begins. Within the SLOs, a specialized microenvironment called the germinal centre (GC) functions as the site of B-cell proliferation and somatic hypermutation, leading to their differentiation into antibody-producing plasma cells or memory B cells (LeBien and Tedder, 2008). In addition to SLOs, GC or GC-like structures have also been described in ectopic or tertiary lymphoid structures (TLSs), where the GC supports local adaptive immune responses towards locally displayed antigens (Pipi *et al.*, 2018).

B cells and the associated microenvironment GCs are vital in mucosal immunity, playing an important role in the protection against the vast array of antigens at the mucosal surface (Vajdy, 2006). In the gastrointestinal and respiratory tracts, B-cell phenotypes and functions have been widely characterized in the associated SLOs, respectively termed the gut-associated lymphoid tissues (GALT) and bronchus-associated or nasopharyngeal-associated lymphoid tissues (BALT or NALT). These secondary lymphoid sites comprise GCs with a distinct B-cell compartment that have a high frequency of surface IgA expressing B cells (Ruddle and Akirav, 2009; McGhee and Fujihashi, 2012), and their dysfunction has been linked with a variety of gastrointestinal and respiratory diseases (Koboziev *et al.*, 2010; Adachi *et al.*, 2015). In non-lymphoid sites in these tissues, such as the lamina propria and epithelial compartments, gut and lung B cells may have unique memory phenotypes and distinct immunoglobulin (Ig) producing capabilities compared with their peripheral counterparts (Brandtzaeg, 2007; Weisel *et al.*, 2020).

The female reproductive tract is also a mucosal system; however, B cells are comparatively understudied. Despite B-cell dysfunction being implicated in benign female reproductive pathologies, such as endometriosis, most studies are focused on peripheral B cells instead of endometrial B cells or relevant tissue B cells (Gagné *et al.*, 2003; Riccio *et al.*, 2017; Danaii *et al.*, 2020). As the innermost lining of the uterus, the human endometrium is a highly dynamic tissue that cyclically sheds and regenerates under the influence of steroid hormones throughout the menstrual cycle, in preparation for embryo implantation during the mid-luteal phase. Therefore, the endometrium not only provides mucosal immune defence against uterine pathogens but also exhibits immune tolerance, as a semi-allogeneic embryo must invade the tissue during placentation for successful pregnancy. At such a dynamic site for immune interactions, B cells were often considered to be rare or absent from the human endometrium (King, 1998; Lee *et al.*, 2011; Schumacher *et al.*, 2018). Recent reviews have not looked into B-cell location, number, phenotype or function in the endometrium, instead, their discussions were mainly based on data from the decidua or peripheral samples, including emerging evidence that cells with a decidual B regulatory phenotype are important during pregnancy (Guzman-Genuino and Diener, 2017; Esteve-Solé *et al.*, 2018; Ticconi *et al.*, 2019).

Nevertheless, some evidence indicates the importance of endometrial B cells in the normal endometrium and endometrium obtained from women with reproductive pathologies: infertility and endometriosis have been associated with a range of autoimmune diseases, which typically result from an expanded population of autoreactive B cells (Hershberg and Luning Prak, 2015; Rawlings *et al.*, 2017; Khizroeva *et al.*, 2019; Shigesi *et al.*, 2019); and endometrial plasma cell levels are often used as a diagnostic criterion for an endometrial inflammatory disorder known as chronic endometritis (CE) (Kitaya *et al.*, 2014; Liu *et al.*, 2018; Cicinelli *et al.*, 2019; Song *et al.*, 2019). In addition, although GC or GC-like structures have not been properly characterized in the normal female reproductive tract, the presence of B-cell centred lymphoid aggregates (LAs) has been widely reported, and these have been suggested to be GC-like, with antigen scavenging and antibody production capacities (Morris *et al.*, 1985; Kitaya and Yasuo, 2010a; Wira *et al.*, 2014; Zhou *et al.*, 2018).

In this systematic review, we provide a comprehensive summary of current evidence investigating B cells in the normal endometrium and benign female reproductive pathologies. It facilitates further understanding of endometrial B cells in the endometrial mucosal immune environment and enhances our understanding of the known link between autoimmune diseases and reproductive pathologies. Here, we discuss the similarities and differences in endometrial B cells in the normal endometrium and various reproductive pathologies, which can serve as the foundation for future studies.

## Materials and methods

This systematic review was registered in PROSPERO in January 2020 (PROSPERO ID: CRD42020152915) (O’Donnell *et al.*, 2020).

### Search strategy

Two independent reviewers conducted the searches (M.S. and E.O.D.). We performed bibliographic searches according to the Preferred Reporting Items for Systematic Reviews and Meta-Analysis (PRISMA) guidelines (Page *et al.*, 2021). The following computerized databases were searched: MEDLINE (via OVID), EMBASE (via OVID), Web of Science Core Collection (Citation Indexes = SCI-EXPANDED, CPCI-S, CPCI-SSH, BKCI-S, ESCI) and CINAHL (via EbscoHost). Each database was searched from inception until 12 November 2021. The search strategy combined the use of subject headings and relevant text words, which included specific terms and free-text terms relating to the endometrium (e.g. ‘endometri*’), B cells (e.g. ‘B cell*’, ‘B lymphocyte*’, ‘plasma cell*’, ‘CD19’, ‘CD20’, ‘CD22’) and possible B-cell derivatives (e.g. ‘antibod*’, ‘immunoglobulin*’). Additionally, non-benign diseases were excluded using free-text terms (e.g. ‘cancer*’, ‘malign*’, ‘tumour*’, ‘carcinoma*’). The searches were limited to publications in English including human subjects only. One additional paper presenting endometrial B-cell data, known to the reviewers but not retrieved in the search, was also included (a peer-reviewed paper investigating the cellular components of human endometrium on the single-cell level) (Lucas *et al.*, 2020)—the paper did not mention the keyword ‘B cells’ in its abstract. All study types were included to maximize the comprehensiveness of the search. A search strategy example for the MEDLINE database is detailed in Supplementary Table SI.

### Inclusion criteria and study selection

Peer-reviewed research papers that studied endometrial B cells in non-pregnant women who were either healthy or presented with benign reproductive pathologies were included in this systematic review. Non-primary research articles, such as reviews, commentaries, correspondence articles, short communications and letters to the editor, were excluded to avoid duplication. Case reports were excluded as their results may not be truly representative of a larger population. We excluded conference abstracts whose respective peer-reviewed full publications were not available to ensure scientific rigour.

Two independent reviewers (M.S. and E.O.D.) carried out the study selection, first by reviewing titles, abstracts and key words to assess paper relevance for endometrial B cells, and then by screening the full text of selected studies identified in the initial phase. Two reviewers then discussed their chosen studies and any discrepancies were resolved through discussion.

### Data extraction

Eligible papers were retrieved and read in detail to extract the following data into a standard form: authors, country, study period, publication year, publication journal, number of participants and demographic data, treatment details (if received), sample obtaining method, sample collection date (menstrual cycle day), sample storage approach, control group selection criteria, diagnostic criteria for gynaecological pathologies (if any), experimental design and reported results.

### Quality assessment

We conducted an assessment of study quality using either the NEWCASTLE-OTTAWA quality assessment scale (Wells *et al.*, 2020) for case–control studies, or the adapted NEWCASTLE-OTTAWA quality assessment scale (Modesti *et al.*, 2016) for cross-sectional studies. Sample selection, group comparability and study outcome were accessed and graded accordingly. If multiple endometrial immune cell types were studied, the quality assessment was based on data related to B cells. Each paper was graded as ‘Good’, ‘Fair’ or ‘Poor’ quality. Two independent reviewers (M.S. and E.O.D.) performed the quality assessment and any disagreements were resolved upon discussion with J.S. and I.G.

## Results

### Study selection

**Figure 1.**
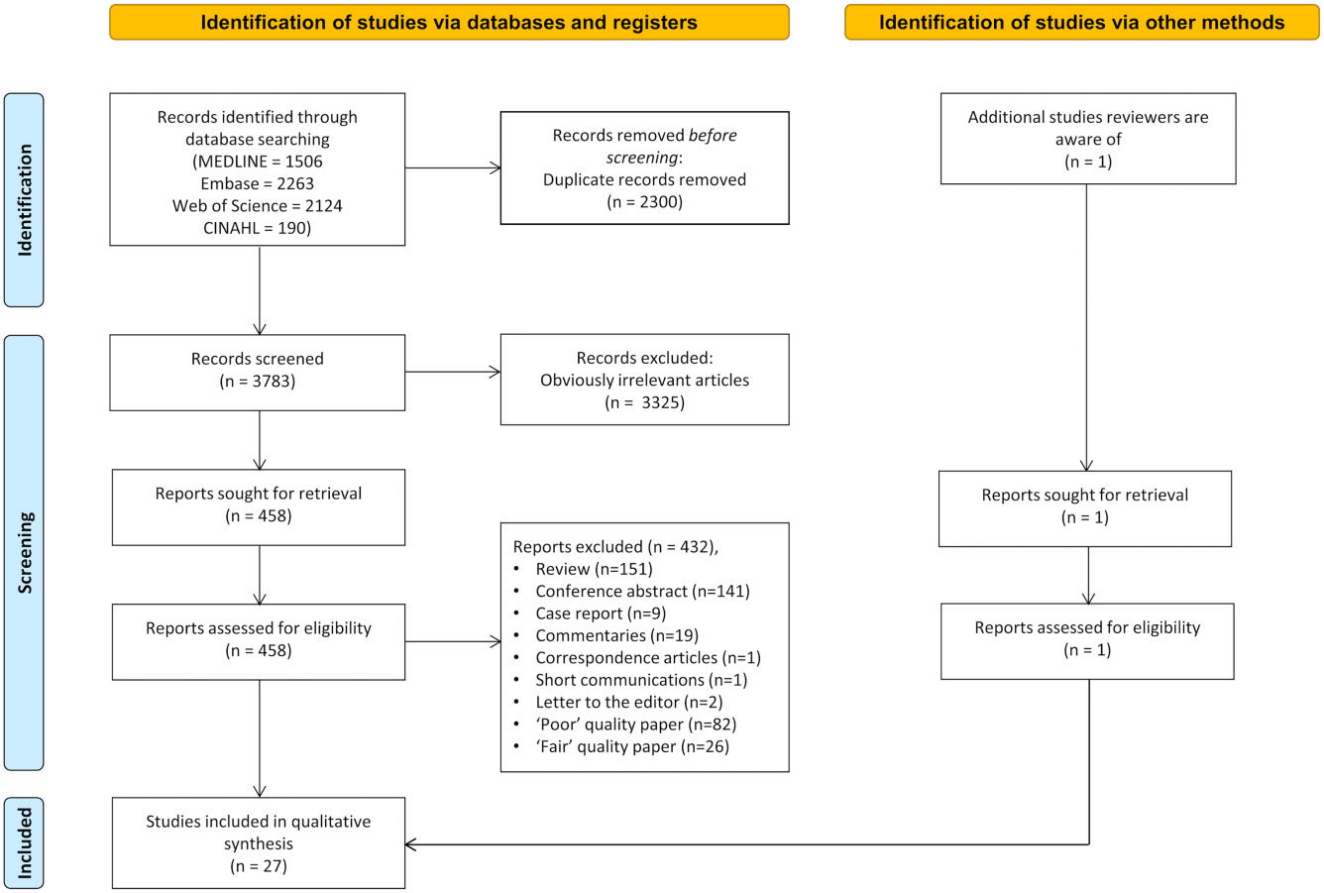
PRISMA flow diagram of study selection.

The database search retrieved 1506 records from MEDLINE, 2263 from Embase, 2124 from Web of Science and 190 from CINAHL. One additional paper known to the reviewers was also included in the identification phase: this paper did not mention B cells or B-cell derivatives in the paper title, abstract or key words, therefore, was not retrieved from database search, but contains endometrial B-cell data in the main text. After de-duplication, in total, 3783 papers were exported for first-phase screening. After reviewing titles, abstracts and key words, 3325 papers were excluded for either not using non-pregnant benign endometrium samples or not investigating B cells. The remaining 459 papers were deemed eligible for the next phase of full-text screening. A total of 324 papers were excluded for not being primary research articles (e.g. reviews, case reports and letters to the editor, Fig. 1). The remaining papers underwent quality assessment, and ‘Poor’ quality papers (n = 82) and ‘Fair’ quality papers (n = 26) were excluded from the qualitative synthesis (Fig. 1). Briefly, a paper was excluded if it met one of the following criteria: no mention of whether participants were pre-menopausal or post-menopausal, or no separation between pre- and post-menopausal women in group analyses; no mention of how B cells were identified, or no specific marker used in B-cell identification; absence of quantitative data, with results only described as endometrial B-cell presence or absence; other significant issue in ‘Sample selection’, ‘Group comparability’ or ‘Study outcome’. Finally, 27 ‘Good’ quality papers were included in the qualitative synthesis. The NEWCASTLE-OTTAWA quality assessment scores for each included study are detailed in Supplementary Table SII.

### B cells in the normal endometrium

The presence and abundance of endometrial B cells and Igs were studied in the normal endometrium in four papers (Table I). Dual-colour flow cytometry showed 3% of all endometrial lymphocytes were CD3^−^CD19^+^ B cells in both the follicular and luteal phases, which was significantly lower than corresponding levels in peripheral blood mononuclear cells (PBMCs) (18%) (Chen *et al.*, 1995). Using immunohistochemistry (IHC), similar proportions of B cells were reported (Klentzeris *et al.*, 1992). Single-cell RNA sequencing analysis of luteal phase endometrium identified a naïve B-cell population in four out of six samples, which were no more than 2% of the total endometrial cell population. Compared with monocytes, macrophage/dendritic cells and natural killer (NK) cells, naïve B cells have a higher expression level of typical B-cell associated genes *CD19, MS4A1* (encoding CD20)*, CD79A*, *LY9* (lymphocyte antigen 9), *CD83*, growth regulator *BTG1* and chemokine receptor *CXCR4* and a moderate level of *CD74* and *HLA-DRA* (Lucas *et al.*, 2020). Mass spectrometry demonstrated the presence of various Igs (heavy chains of IgG, IgM, IgJ, IgA1, IgA2 and light chain of Igκ) in the human endometrium, although they were less abundant in the endometrium than in the endocervix and ectocervix (Burgener *et al.*, 2013).

**Table I:** Studies examining B cells in the normal endometrium.

Citation	Study type	Participant information			Menstrual cycle phase of the samples obtained	Hormone treatment	Experimental method	Marker for B cell (subset)	Key findings
		Age (years)	Number	Characteristics					
Burgener *et al.* (2013)	Cross-sectional	42–57	7	Without malignant and inflammatory conditions, HIV IgG seronegative and no clinical symptoms of sexually transmitted infections during the 3 months prior to endometrial sample collection	–	None were on hormonal contraceptives	Mass spectrometry	heavy chains of IgG, IgM, IgJ, IgHA1, IgHA2 and light chain of Igκ	Various immunoglobulins (heavy chains of IgG, IgM, IgJ, IgA1, IgA2 and light chain of Igκ) were present in human endometrium.
							IHC	IgA	IgA was mainly located at or adjacent to the columnar endometrium epithelium.
Klentzeris *et al.* (1992)	Cross-sectional	20–40	24	Had one or more successful pregnancies and a regular menstrual pattern, without endometrial pathology, pelvic endometriosis or anatomic abnormalities of the uterus, no coitus for over 48 h before the endometrial sample collection	Luteal phase	No steroid hormone treatment 3 months before biopsy collection	IHC	CD22	B cells represent 2–3% of total CD45+ leucocytes, were detected in stroma as well as lymphoid aggregates.
Chen *et al.* (1995)	Cross-sectional	Mean age 42–43	39	Without malignant condition or endometrial pathology, no evidence of infection or inflammation	Follicular phase and luteal phase	–	Flow cytometry	CD19	Significantly lower B-cell percentage in endometrium (3%) compared with PBMCs (18%).
Lucas *et al.* (2020)	Cross-sectional	31–42	6	With regular menstrual cycles, BMI between 23 and 32 kg/m^2^, and absence of uterine pathology on transvaginal ultrasound examination	Mid-luteal phase (LH + 8) and late-luteal phase (LH + 10)	None	Single-cell RNA-seq	–	Naïve B cells, with no more than 2% of total endometrial cell population, were detected in human endometrium.

IHC, immunohistochemistry; PBMC, peripheral blood mononuclear cell.

**Figure 2.**
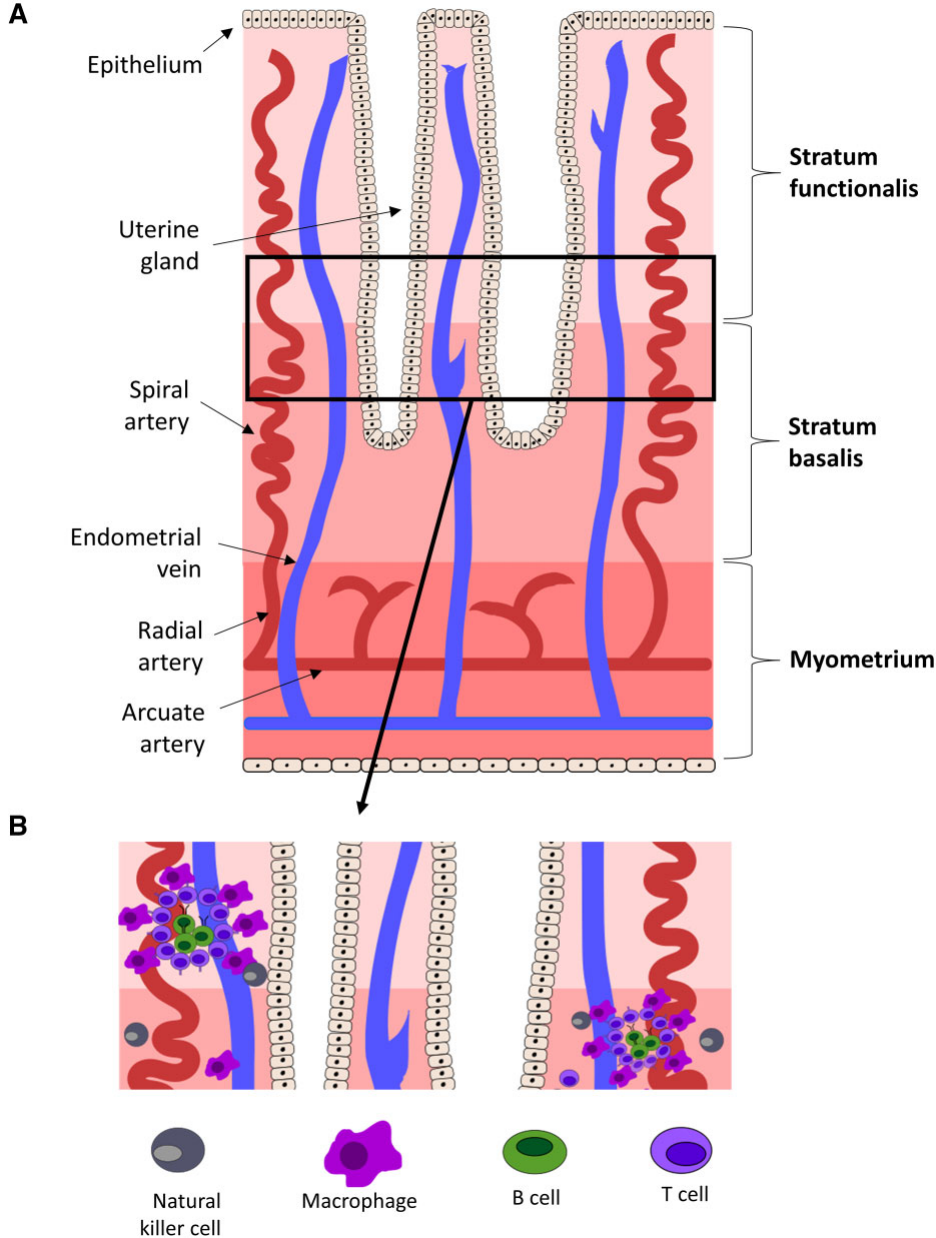
**Illustration of lymphoid aggregates in human endometrium**. (**A**) The uterine lining, including the stratum functionalis and basalis (the endometrium), and the underlying myometrium. (**B**) A close-up of the lower functionalis and the basalis layers of the endometrium. The lymphoid aggregates are composed of a B-cell core, surrounded by a circle of T cells and a halo of macrophages. Natural killer cells are scattered throughout the stroma.

In terms of location, CD22^+^ B cells were found scattered within endometrial stroma as well as in LAs (Fig. 2) (Klentzeris *et al.*, 1992). Evidence of LA-associated B cells in the normal endometrium was found in the control groups of case–control studies (Klentzeris *et al.*, 1994, 1995; Fernández-Shaw *et al.*, 1995; Mettler *et al.*, 1997; Disep *et al.*, 2004; Kitaya and Yasuo, 2010a). Apart from B cells (identified by CD20 or CD22), these aggregates also consist of other CD45^+^ cells, including CD3^+^ T cells, CD4^+^ T cells, CD68^+^ macrophages and CD57^+^ NK cells, with B cells in the central area and T cells and macrophages in the margin areas (Klentzeris *et al.*, 1992; Mettler *et al.*, 1997; Disep *et al.*, 2004). LAs were shown to be comprised of several hundred cells, express the endometrial proliferation marker Ki-67 (examined by Ki-S3 antibody) and were mostly HLA-DR^+^ (Mettler *et al.*, 1997). They were situated in the lower functional layer, or the basal layer, and were observed throughout the menstrual cycle (Fernández-Shaw *et al.*, 1995; Mettler *et al.*, 1997). Additionally, LAs appeared to have a cyclical influence and were more likely to appear in the follicular phase of the menstrual cycle (Mettler *et al.*, 1997). Additionally, IgA was found mainly at or adjacent to the columnar endometrium epithelium (Burgener *et al.*, 2013).

### Endometriosis

Endometriosis is defined as the growth of endometrial-like tissue outside of the uterus (Zondervan *et al.*, 2020). Endometrial B cells and Igs have been investigated in endometriosis in four studies (Table II). Methods for detection were either flow cytometry or IHC, and no single-cell RNA sequencing studies focusing on endometrial B-cell populations have been performed to date. One study using dual-colour flow cytometry showed that CD20^+^ B cells were significantly more abundant in 20 endometriosis samples compared with 15 controls; 10% of the lymphocytes (identified by CD45^+^CD14^−^) were CD20^+^ endometrial B cells in controls, 12% in eutopic endometrium and 17% in ectopic endometrium from patients with endometriosis. Moreover, CD20^+^ B cells in ectopic endometrium had significantly elevated CD5 and HLA-DR expression levels compared with the eutopic endometrium in both patients with endometriosis and controls (Antsiferova *et al.*, 2005). Using CD22, three IHC studies reported similar endometrial B-cell counts between, in total, 169 endometriosis patients and 138 controls (Fernández-Shaw *et al.*, 1995; Klentzeris *et al.*, 1995; Mettler *et al.*, 1997). Specifically, Klentzeris *et al.* (1995) reported that B cells represented 3–4% of total CD45^+^ cells in all samples: B cells were scattered throughout the stroma with a few seen in LAs. Mettler *et al.* (1997) showed B cells were almost exclusively detected in LAs, and the maximal quantity was present in the early follicular phase (40 CD22^+^ cells per mm^2^), then decreased during the mid-follicular phase and remained relatively constant in the luteal phase (20 CD22^+^ cells per mm^2^) in all samples. Fernández-Shaw *et al.* (1995) reported, on average, 4-5 CD22^+^ cells per 989 µm^2^ (cell count ranging from 0 to 40 per 989 µm^2^) in all samples during both the follicular and luteal phases.

**Table II:** **Studies examining endometrial B cells in controls and patients with endometriosis**. Number of participants

**Citation**	**Study type**	**Control group**		**Endometriosis group**		**Age (years)**	**Menstrual cycle phase of the samples obtained**
					
		**Control characteristics**	**Number of participants**	**Endometriosis diagnosis**	**Number of participants**		

Fernández-Shaw *et al.* (1995)	Case–control	Regular but heavy menstrual cycles and no evidence of pelvic disease	10	Laparoscopically diagnosed, diagnosis and classification of endometriosis was based on the revised criteria of the American Fertility Society (The American Fertility Society, 1985)	8	Premenopausal	Variable
Antsiferova *et al.* (2005)	Case–control	Healthy women with previously documented fertility and no relevant gynaecological history	15	Laparoscopically diagnosed	20	21–40	–
Klentzeris *et al.* (1995)	Case–control	No endometriosis, 1 or more successful pregnancies in the past, with regular menstrual cycles	18	Infertile women^a^ who had laparoscopically diagnosed endometriosis. Diagnosis and classification of endometriosis was based on the revised criteria of the American Fertility Society (The American Fertility Society, 1985)	18	22–41	Early-, mid- and late-luteal phase
Mettler *et al.* (1997)	Case–control	Laparoscopically proven tubal factor infertility resulting from prior pelvic inflammatory disease. No signs of endometrial inflammation	110	Laparoscopically diagnosed, diagnosis and classification of endometriosis was based on the revised criteria of the American Fertility Society (The American Fertility Society, 1985)	143	Mean age, 30	Variable
Holzer *et al.* (2021)	Cross-sectional	–	–	Diagnosis and classification of endometriosis was based on rASRM (Johnson *et al*., 2017)	55	18–44	Variable, mostly (87%) in the follicular phase
**Citation**	**Hormone treatment**	**Experimental method**	**Marker for B cell (subset)**	**Key findings**			

Fernández-Shaw *et al.* (1995)	None	IHC	CD22	B cells were detected in stroma as well as lymphoid aggregates. Similar B-cell count in control and endometriosis group (on average 4–5 CD22^+^ cells per 989 µm^2^)			
Antsiferova *et al.* (2005)	None within last 3 months	Flow cytometry	CD20 (HLA-DR, CD5)	Significantly higher (*P* < 0.05) B-cell percentage in endometriosis group (12% of all lymphocytes in eutopic endometrium and 17% in ectopic endometrium) compared with controls (10% of all lymphocytes)			
Klentzeris *et al.* (1995)	None when sample was collected	IHC	CD22	B cells were detected in stroma as well as lymphoid aggregates. Similar B-cell count in control and endometriosis group (3–4% of total CD45^+^ cells)			
Mettler *et al.* (1997)	–	IHC	CD22	B cells were exclusively detected in lymphoid aggregates. Similar B-cell count in control and endometriosis group (20 CD22^+^ cells per mm^2^)			
Holzer *et al.* (2021)	–	IHC	CD138	CD138^+^ plasma cell count per 20 high-power field ranging from 0 to 13, and the cell count was positively correlated with the endometriosis rASRM stage (r = 0.302, *P* = 0.028)			

rASRM, revised American Society for Reproductive Medicine.

a As detailed in Table III, infertility is diagnosed when participants were unable to conceive after at least 12 months of unprotected intercourse (Klentzeris *et al.*, 1995).

Additionally, plasma cells have been identified as CD138^+^ in patients with endometriosis. In a study by Holzer *et al.*, a weakly positive correlation was observed between the mean number of CD138^+^ plasma cells and the revised American Society for Reproductive Medicine (rASRM) stage of endometriosis (r = 0.302, *P* = 0.028). All samples had CD138^+^ plasma cell counts (per 20 high-power fields) ranging from 0 to 13, and women with endometriosis in advanced stages were more likely to have an increased count of CD138^+^ plasma cells (Holzer *et al.*, 2021).

### Infertility, recurrent implantation failure and recurrent pregnancy loss

Endometrial B cells, plasma cells and Igs were investigated in nine studies in women with a history of infertility, recurrent implantation failure (RIF) and recurrent pregnancy loss (RPL) compared to healthy controls. The diagnostic criteria for RPL, RIF and infertility vary between different papers (Table III). One study used flow cytometry to investigate endometrial B cells (Lachapelle *et al.*, 1996), eight studies used only IHC (Klentzeris *et al.*, 1994, 1995; Quenby *et al.*, 1999; Michimata *et al.*, 2002; Kitaya *et al.*, 2014; Liu *et al.*, 2018, 2018; Fan *et al.*, 2021) and one study combined IHC and an RNase protection assay (Bohlmann *et al.*, 2010). No study has conducted single-cell RNA sequencing to investigate B-cell populations in relation to infertility, RIF or RPL.

**Table III:** Studies examining endometrial B cells in patients with infertility, RIF and RPL.

Citation	Study type	Control group		Reproductive pathologies group			Age (years)	Menstrual cycle phase of the samples obtained
		Control characteristics	Number of participants	Participants’ selection		Number of participants		
Michimata *et al.* (2002)	Case–control	Successful pregnancy after IVF treatment for solely male factor infertility	15	A history of two consecutive first-trimester miscarriages, with subsequent IVF treatment outcome	Successful pregnancy	11	Mean age, 30–32	Mid-luteal phase (day 5–8 after LH surge)
					Pregnancy loss (RPL)	6		
Lachapelle *et al.* (1996)	Case–control	Healthy fertile women	15	A history of 3 or more unexplained, consecutive, first trimester spontaneous miscarriages with the same partner	Primary RPL	11	22–40	Luteal phase
					Secondary RPL	9		
Quenby *et al.* (1999)	Case–control	Two or more normal pregnancies	9	At least three consecutive miscarriages (RPL)		22	20–41	Mid-luteal phase
Bohlmann *et al.* (2010)	Case–control	–	10	17 Women had 3 or more idiopathic early miscarriages, and 8 women had 2 early miscarriages (RPL)		25	21–41	Luteal phase
McQueen *et al.* (2021)	Case–control	Women without a history of RPL, infertility (inability to conceive for more than 1 year)	26	Two or more pregnancy losses (unexplained RPL)		50	Mean age: 33.3 (controls), 35.2 (RPL)	–
Kitaya *et al.* (2014)	Case–control	Proven fertile women	22	Consecutive negative pregnancy tests following transfer of three or more morphologically good embryos and/or blastocysts (RIF)	Non-endometritis patients^a^	23	Mean age, 37	Follicular phase
					Endometritis patients^a^	28		
Li *et al.* (2021)	Cross-sectional	–	–	Infertile patients who received IVF-embryo transfer treatment with an average of 3 years infertility		716	<45	Mid-luteal phase
Klentzeris *et al.* (1995)	Case–control	One or more successful pregnancies in the past	18	Endometriosis patients^b^ who are unable to conceive alter at least 12 months of unprotected intercourse (infertility)		18	22–41	Early, mid and late luteal phase
Klentzeris *et al.* (1994)	Case–control	One or more successful pregnancies	24	A history of involuntary infertility of >2 years (unexplained infertility)		24	24–43	Luteal phase
Fan *et al.* (2021)	Cross-sectional	–	–	Patients with 2–4 years of infertility and failed with IVF fresh cycle in which at least 2 high-quality embryos were transferred		78	20–38	Mid-luteal phase
Zargar *et al.* (2020)	Cross-sectional	–	–	Failure to achieve a clinical pregnancy after two good-quality embryo transfers in fresh or frozen cycles (RIF)		47	Mean age, 36	Follicular phase (3–5 days after menstruation)
				Two or more consecutive pregnancy losses or three or more alternate pregnancy losses (<20 weeks of gestational ages) (RPL)		38		
Liu *et al.* (2018)	Case–control	At least one live birth within the previous 2 years	40	Loss of three or more consecutive pregnancies before 24 weeks of gestation (RPL)		93	20–40	Mid-luteal phase
				Fail to achieve clinical pregnancy after transfer of at least four good-quality embryos in three or more transfer cycles (RIF)		39		
				Infertility		48		

Citation	Hormone treatment	Experimental method	Marker for B cell (subset)	Key findings
Michimata *et al.* (2002)	–	IHC	CD20	Similar B-cell count in control and RPL group (2–3% of all endometrial CD45^+^ cells)
Lachapelle *et al.* (1996)	–	Flow cytometry	CD20	Significant increase in CD20^+^ endometrial B cells in RPL (16 ± 8% of CD45^+^ cells) when compared with normal controls (5 ± 6% of CD45^+^ cells). Similar B-cell percentages in primary and secondary RPL.
Quenby *et al.* (1999)	–	IHC	CD22	Similar B-cell count in control and RPL group (0–4% of total endometrial cells)
Bohlmann *et al.* (2010)	None within last 3 months	IHC and RNase protection assay	CD19	Similar B-cell count in control and RPL group
McQueen *et al.* (2021)	–	IHC	CD138	CD138^+^ plasma cells were identified in both control and RPL groups, no significant difference was observed in terms of the patient proportion with ≥1, 2 or 5 CD138^+^ plasma cells per 10 high-power fields
Kitaya *et al.* (2014)	Controlled ovarian stimulation protocol in most RIF patients	IHC	CD138, IgM, IgA1, IgA2, IgG1, IgG2, IgG3, IgG4, IgD, IgE	Significant increase in IgM^+^ stromal cell density in RIF group (regardless of endometritis diagnosis) compared with controls
Li *et al.* (2021)	–	IHC	CD138	In patients with ≥5 endometrial CD138^+^ plasma cells per high-power field, β-hCG positive rate, clinical pregnancy rate and live birth rate were significantly lower than that in patients with <5 endometrial CD138^+^ plasma cells. No significantly difference on pregnancy outcome was observed between patients with no CD138^+^ plasma cells and patients with up to four plasma cells per high-power fields
Klentzeris *et al.* (1995)	None when sample was collected	IHC	CD22	B cells were detected in stroma as well as lymphoid aggregates. Similar B-cell count in control and infertility group (constituted 3–4% of all endometrial CD45^+^ cells)
Klentzeris *et al.* (1994)	–	IHC	CD22	B cells were detected in stroma as well as lymphoid aggregates. Similar B-cell count in control and infertility group (constituted 2–3% of all endometrial CD45^+^ cells)
Fan *et al.* (2021)	Received controlled ovarian hyperstimulation	IHC	CD19	B cells were detected in the mid-luteal phase endometrium, with an average of 0.34% CD19^+^ B cells in all stromal cells
Zargar *et al.* (2020)	–	IHC	CD138^c^	36.8% (14 subjects) of patients with RPL and 23.4% (11 subjects) of patients with RIF had over 5 plasma cells per 20 high-power fields in endometrial samples
Liu *et al.* (2018)	None with in last 2 months	IHC	CD138^c^	Similar B-cell count in control and infertility, RIF and RPL groups (most samples have CD138^+^ cell counts <5 per 0.1 mm^2^)

RIF, recurrent implantation failure; RPL, recurrent pregnancy loss.

^a^As detailed in Table IV, endometritis was diagnosed when there were unusual plasmacyte infiltrates within the endometrial stromal compartment (Kitaya *et al.*, 2014).

^b^As detailed in Table II, endometriosis was diagnosed laparoscopically, according to the rASRM criteria (The American Fertility Society, 1985; Klentzeris *et al.*, 1995).

^c^CD138 staining was performed as a diagnostic criterion for chronic endometritis.

Investigating women with a history of infertility, no significant difference in endometrial CD22^+^ B-cell numbers was found by IHC in two studies between, in total, 42 control and 42 infertile patients (Klentzeris *et al.*, 1994, 1995). CD22^+^ B cells constituted 2–4% of all endometrial CD45^+^ cells and were present in both stroma and LAs (Klentzeris *et al.*, 1994, 1995). CD138^+^ plasma cells were also identified in infertile patients. In 716 infertile patients that underwent IVF-embryo transfer, a significantly higher pregnancy rate (β-hCG positive rate, clinical pregnancy rate and live birth rate) was observed in patients with <5 endometrial CD138^+^ plasma cells per high-power field compared with patients that have ≥5 endometrial CD138^+^ plasma cells per high-power fields. No significant difference in pregnancy outcome was observed between patients with no CD138^+^ plasma cells and patients with up to four plasma cells per high-power field (Li *et al.*, 2021).

In patients with RPL, the flow cytometry study reported a significant increase in CD20^+^ endometrial B cells compared with normal controls during the luteal phase (5 ± 6% of CD45^+^ cells in controls versus 16 ± 8% of CD45^+^ cells in RPL) (Lachapelle *et al.*, 1996). B-cell percentages were similar between primary and secondary RPL and no correlation was observed between B-cell percentages and the number of pregnancy losses (Lachapelle *et al.*, 1996). Two IHC studies using either CD20 or CD22 as a B-cell marker revealed no significant difference in endometrial B-cell numbers between controls and women with RPL (Quenby *et al.*, 1999; Michimata *et al.*, 2002). Specifically, Michimata *et al.* (2002) found similar levels of CD20^+^ endometrial B cells (2–3% of all endometrial CD45^+^ cells) in controls and RPL patients, regardless of their subsequent pregnancy outcome. Quenby *et al.* (1999) reported CD22^+^ B cells comprised 0–4% of total endometrial cells (per 10 high power fields); of note, 2 (out of 31) women had an elevated B-cell count, and in the pregnancy following endometrium analysis, one patient had a live birth and the other had a miscarriage. Using CD19 as the B-cell marker, combined with an RNase protection assay for validation, Bohlmann *et al.* (2010) also showed no significant difference in B-cell numbers between healthy women and those with RPL. In a recent cross-sectional IHC study that included 78 infertile patients, Fan *et al.* (2021) reported an average of 0.34% CD19^+^ B cells in all stromal cells. Apart from pan-B-cell markers, such as CD19, CD20 and CD22, a recent study investigated the presence of CD138^+^ plasma cells in patients with RPL versus controls. CD138^+^ plasma cells were identified in both control and RPL groups, with no significant difference observed between patients and controls when analysing ≥1, 2 or 5 CD138^+^ plasma cells per 10 high-power fields (McQueen *et al.*, 2021).

For RIF, Ig content was also analysed by IHC in a single study. There was a significant increase in IgM^+^ stromal cell density during the follicular phase in patients with RIF (regardless of endometritis diagnosis) compared with controls (Kitaya *et al.*, 2014).

Additionally, two studies included more than one patient group. Using IHC for CD138^+^ to identify plasma cells, one cross-sectional study found 36.8% (14 subjects) of patients with RPL and 23.4% (11 subjects) of patients with RIF had over 5 plasma cells per 20 high-power fields in endometrial samples (Zargar *et al.*, 2020). One case–control study reported no significant difference in plasma cell counts when comparing different subgroups, including controls, RPL, RIF and infertile groups, and most samples had CD138^+^ cell counts <5 per 0.1mm^2^ (Liu *et al.*, 2018).

### Endometritis

Endometritis is defined as the inflammation of the lining of the uterus and may be acute or chronic in presentation. Acute endometritis is manifested with fever, pelvic pain and vaginal discharge. CE, on the other hand, is usually asymptomatic or oligosymptomatic. CE has been linked with adverse reproductive outcomes including RIF and RPL (Huang *et al.*, 2020). Our search retrieved one paper that focused on endometritis (acute and chronic) (Disep *et al.*, 2004) and seven papers focused solely on CE (Toth *et al.*, 2007; Kitaya and Yasuo, 2010a; Kitaya *et al.*, 2014; Liu *et al.*, 2018; Parks *et al.*, 2018; Cicinelli *et al.*, 2019; Song *et al.*, 2019). In the included papers, there was no agreed definition or test for the diagnosis of CE, and both hysteroscopic examination and histopathological assessment have been used. In histopathological assessment, CE was confirmed by the presence of plasma cells, identified by markers, such as CD138 and multiple myeloma oncogene 1 (MUM1), or morphological assessment after standard histological staining with hematoxylin phloxine saffron stain (HPS) or hematoxylin and eosin (H&E) (Table IV). Using different diagnostic methods to identify B cells, plasma cells or Igs, the reported prevalence of CE varied in the same cohort (Toth *et al.*, 2007; Liu *et al.*, 2018; Parks *et al.*, 2018; Cicinelli *et al.*, 2019; Song *et al.*, 2019; Zargar *et al.*, 2020).

**Table IV:** Studies examining endometrial B cells in patients with endometritis.

Citation	Study type	Control group		Endometritis group			Age (years)	Menstrual cycle phase of the samples obtained
		Control characteristics	Number of participants	Endometritis diagnosis		Number of participants		
Kitaya and Yasuo (2010a)	Case–control	No sign of plasma cells by CD138 staining in endometrial samples	54	CD138^+^ punctate staining in endometrial samples		22	Premenopausal	Mid-luteal phase
Toth *et al.* (2007) ^a^	Case–control^a^	–	–	Surgical diagnosis		30 out of 92	16–46	–
				HPS staining and morphological plasma cell identification	in endometrial samples	38 out of 92		
				H&E staining and morphological plasma cell identification		72 out of 92		
				CD138 and HPS staining to find plasma cells		76 out of 92		
Liu *et al.* (2018) ^b^	Case–control^b^	–	–	1 or more plasma cells per 10 per 10 high power fields	in endometrial samples, identified by CD138	12 out of 220	20–40	Mid-luteal phase
				1.95 or more plasma cells per 10 per 10 high power fields		36 out of 220		
				1 or more plasma cells per whole section		95 out of 220		
				2.95 or more plasma cells per whole section		42 out of 220		
				5.15 or more plasma cells per 0.1 mm^b^		20 out of 220		
Cicinelli *et al.* (2019) ^c^	Case–control^c^	–	–	Hysteroscopic examination based on the demonstration of micro-polyps, polypoid endometrium, stromal oedema and focal or diffuse hyperaemia		238 out of 480	Mean age, 33	Follicular phase
				1–5 plasma cells per high-power field or discrete clusters of <20 plasma cells endometrial samples identified by CD138		206 out of 480		
Song *et al.* (2019)	Cross-sectional	–	–	1 or more plasma cell identified per 10 high power fields in endometrial samples, identified by CD138		322 out of 1189	Mean age, 33	Follicular phase
				Endometrium with one or more hysteroscopic features, hyperaemia, micro-polyps, and/or interstitial oedema		454 out of 1189		
Zargar *et al.* (2020)	Cross-sectional	–	–	More than 5 plasma cells per 20 high-power fields in endometrial samples identified by CD138	RPL patients^d^	14 out of 38	Mean age, 36	Follicular phase (3 to 5 days after menstruation)
					RIF patients^d^	11 out of 47		
				Hysteroscopy examination, direct observation of mucosal oedema, focal or diffuse endometrial hyperaemia, and the presence of micropolyps (<1 mm)	RPL patients^d^	12 out of 38		
					RIF patients^d^	10 out of 47		
Parks *et al.* (2018)	Cross-sectional	–	–	1 or more plasma cells in endometrial samples, identified by MUM1, CD138, and H&E		55 out of 87	Median age, 36	Variable
				1 or more plasma cells in endometrial samples, identified by MUM1 and H&E		118 out of 224		
Disep *et al.* (2004)	Case–control	Histologically normal, no evidence of any endometrial pathology	22	Based on the presence of endometrial stromal plasma cells and/or neutrophils, inflammation was graded as:	Mild	25	Variable	Variable
					Moderate	32		
					Severe	22		
Kitaya *et al.* (2014)	Case–control	No CE, proven fertile women	22	RIF patients^e^ with unusual plasmacyte infiltrates within the endometrial stromal compartment (histopathologic diagnosis)		28	Mean age, 37	Follicular phase
		No CE, consecutive negative pregnancy tests following transfer of 3 or more morphologically good embryos	23					

Citation	Hormone treatment	Experimental method	Marker for B cell (subset)	Key findings
Kitaya and Yasuo (2010a)	–	IHC	CD138, CD20	B cells were detected in stroma as well as lymphoid aggregates in all samples. In CE, B cells were seen in greater numbers (around 0.41 B cells in controls vs 47 B cells per 20 gland lumina in CE) and unusual locations. B-cell aggregates were present with an increased size in CE.
Toth *et al.* (2007)	65% of participants named oral contraception as their primary method of birth control	IHC	CD138	More CE cases were reported using histopathological assessment compared with hysteroscopic examination. Significant correlation was reported between higher plasma cell counts (>5) and *Chlamydia trachomatis* infection.
Liu *et al.* (2018)	None with in last 2 months	IHC	CD138	Numbers of CE cases were different when different numbers of plasma cells were used as diagnostic criteria.
Cicinelli *et al.* (2019)	None within last 3 months	IHC	CD138	More CE cases were reported using hysteroscopic examination compared with histopathological assessment.
Song *et al.* (2019)	–	IHC	CD138	More CE cases were reported using hysteroscopic examination compared with histopathological assessment.
Zargar *et al.* (2020)	–	IHC	CD138	More CE cases were reported using histopathological assessment compared with hysteroscopic examination.
Parks *et al.* (2018)	–	IHC	CD138, MUM1	In histopathological assessment, CE prevalence was different when different markers were used for plasma cells identification.
Disep *et al.* (2004)	–	IHC	CD20	B cells were detected in stroma as well as lymphoid aggregates in all samples. In endometritis, B cells were seen in greater numbers (<1% of CD45^+^ cells in controls vs up to 25% in endometritis) and unusual locations. The sizes of B-cell aggregates were increased with endometrial inflammation severity.
Kitaya *et al.* (2014)	Controlled ovarian stimulation protocol in most RIF patients	IHC	CD138, IgM, IgA1, IgA2, IgG1, IgG2, IgG3, IgG4, IgD, IgE	IgM^+^ has the highest density among all assessed immunoglobulins. Significantly higher IgM^+^, IgA1^+^, IgA2^+^, IgG1^+^ and IgG2^+^ densities in CE patients with RIF compared with both RIF patients without CE and controls.

HPS, haematoxylin phloxine saffron stain; H&E, haematoxylin and eosin; MUM1, multiple myeloma oncogene 1.

a A case–control study on *C. trachomatis* and abnormal uterine bleeding. In total 92 cases, *C. trachomatis* was detected in 44 cases, abnormal uterine bleeding was diagnosed in 65 cases (as detailed in Table V), uterine fibroids and ovarian cysts were noted in 27 cases (Toth *et al.*, 2007).

b A case–control study on infertility, RIF and RPL patients (as detailed in Table III), in total 220 cases, 40 were controls, 93 were RPL patients, 39 were RIF patients and 48 were infertile patients (Liu *et al.*, 2018).

c A case–control study on endometrial polyps (as detailed in Table V), in total 480 cases, 50% of all participants had endometrial polyps (Cicinelli *et al.*, 2019).

d In total, 38 RPL patients and 47 RIF patients were included. As detailed in Table III, RPL was diagnosed when participants had 2 or more consecutive pregnancy losses or three or more alternate pregnancy losses (<20 weeks of gestational ages), RIF was diagnosed when participants failed to achieve a clinical pregnancy after two good-quality embryo transfers in fresh or frozen cycles (Zargar *et al.*, 2020).

e As detailed in Table III, RIF was diagnosed when participants had consecutive negative pregnancy tests following transfer of three or more morphologically good embryos and/or blastocysts (Kitaya *et al.*, 2014).

Endometrial B cells, plasma cells and Ig contents have been studied in endometritis by IHC (Table IV). Compared with controls, where CD20^+^ B cells are found in the basal and stromal layers, in patients with endometritis they were more abundant and in alternate locations, such as in the epithelium and glandular lumina (Disep *et al.*, 2004; Kitaya and Yasuo, 2010a). Disep *et al.* (2004) showed CD20^+^ B cells comprised fewer than 1% of all CD45^+^ cells in the normal endometrium but up to 25% in endometritis samples. Kitaya and Yasuo (2010a) showed that, on average, there were 0.41 CD20^+^ B cells per 20 gland lumina in controls and 47 B cells per 20 gland lumina in patients with CE. Additionally, CD20^+^ B-cell counts also increased with endometrial inflammation severity (graded according to the presence of plasma cells and neutrophils) along with the size of LAs (Disep *et al.*, 2004). In CE, CD138^+^ plasma cells counts (>5) were statistically correlated with *Chlamydia trachomatis* infection (Toth *et al.*, 2007). The densities of IgM^+^, IgA1^+^, IgA2^+^, IgG1^+^ and IgG2^+^ during the follicular phase were significantly higher in patients with CE and RIF compared to both patients with RIF but without CE and healthy controls. Among all five types of Igs assessed, IgM^+^ had the highest density, followed by IgG2^+^, IgA1^+^ and IgA2^+^, with each one significantly lower than the former. IgG1^+^ density was significantly lower than IgG2^+^, but significantly higher than IgA2^+^ (Kitaya *et al.*, 2014).

### Other pathologies

CD20^+^ endometrial B cells and CD138^+^ endometrial plasma cells were also studied by IHC in patients suffering from abnormal uterine bleeding, endometrial polyps and uterine fibroids (Table V). The CD20^+^ B-cell density was low in both 24 controls and 62 patients with uterine fibroids (0–3 in 10 non-overlapping stromal areas) and no difference was observed throughout the menstrual cycle (Kitaya and Yasuo, 2010b). Toth *et al.* (2007) examined the correlation between abnormal uterine bleeding and *C. trachomatis* CE (diagnosed by CD138^+^ staining) and reported CD138^+^ plasma cells were present in 80% of 65 patients with abnormal uterine bleeding, compared to 41% in 27 controls. A greater prevalence of CD138^+^ plasma cells in the follicular phase was reported in a large study of 240 patients with endometrial polyps compared with the 240 controls (62% vs 24%, respectively). Moreover, 77% of endometrial polyps had CD138^+^ staining, and within this group, 64% also had CD138^+^ staining on their corresponding endometrial samples. Whereas only 30% of CD138− endometrial polyps coexist with a CD138^+^ endometrium (Cicinelli *et al.*, 2019).

**Table V:** Studies examining endometrial B cells in controls and patients diagnosed with abnormal uterine bleeding, endometrial polyps and uterine fibroids.

Citation	Study type	Control group			Pathological group			Age (years)	Menstrual cycle phase of the samples obtained
		Control characteristics		Number of participants	Pathology and patients’ characteristic (if stated)		Number of participants		
Toth *et al.* (2007) ^a^	Case–control	Without abnormal uterine bleeding diagnosis		27	Abnormal uterine bleeding		65	16–46	–
Cicinelli *et al.* (2019)	Case–control	Normal endometrium without evidence of endometrial polyps at hysteroscopy^b^		240	Endometrial polyps, diagnosed at hysteroscopy and histology, identified as localized overgrowths of endometrial mucosa		240^c^	Mean age, 33	Follicular phase
Kitaya and Yasuo (2010b)	Case–control	Normal endometrium without uterine fibroids	With benign ovarian tumours	20	Uterine fibroids, classified into three categories according to the location within the uterine wall:	Submucosal, causing cavity distortions	24	37–45	Variable
			With uterine prolapse	4		Intramural, with <50% extension outside the myometrium without any cavity distortions	25		
						Subserosal, with 50% or more extension outside the myometrium without cavity distortions	13		

Citation	Hormone treatment	Experimental method	Marker for B cell (subset)	Key findings
Toth *et al.* (2007)	65% of participants named oral contraception as their primary method of birth control	IHC	CD138	Greater prevalence of plasma cell presence was reported in patients with abnormal uterine bleeding compared with controls (80% vs 41%).
Cicinelli *et al.* (2019)	None within last 3 months	IHC	CD138	Greater prevalence of plasma cell presence was reported in patients with endometrial polyps compared with controls (62% vs 24%).
Kitaya and Yasuo (2010b)	None	IHC	CD20	Similar B-cell count (0–3 in 10 non-overlapping stromal areas) in patients with uterine fibroids and controls.

^a^In total 92 cases, *C. trachomatis* was detected in 44 cases, uterine fibroids and ovarian cysts were noted in 27 cases. Additionally, CE was diagnosed by H&E, HPS and/or CD138 staining, as detailed in Table IV (Toth *et al.*, 2007).

^b^In total 240 cases, 136 women had a submucous/intramural uterine myoma. Additionally, CE was diagnosed by hysteroscopic examination or CD138 staining, as detailed in Table IV (Cicinelli *et al.*, 2019).

^c^About 240 endometrial polyps’ samples from the same group of patients were also collected and analysed (Cicinelli *et al.*, 2019).

## Discussion

This systematic review is the first to summarize the evidence for endometrial B-cell location, number, phenotype and function in normal endometrium and in benign endometrial pathologies, with a comprehensive searching and grading process. Although there have been a few reviews on the role of B cells in gynaecological conditions, these articles have mostly discussed blood-based B cells and antibody titres in one specific pathology, without a quality assessment of the endometrial B-cell data included (Muzzio *et al.*, 2013; Riccio *et al.*, 2017; Vallvé-Juanico *et al.*, 2019).

We included 17 case–control and 10 cross-sectional studies in this systematic review; endometrial B cells were studied in healthy participants and women with endometriosis, infertility, RIF, RPL, endometritis, abnormal uterine bleeding, endometrial polyps and uterine fibroids. Six studies had mixed patient populations with more than one diagnosed condition, mostly to evaluate the prevalence of CE or endometriosis in patients with RPL. The experimental methods used were IHC alone (n = 21), flow cytometry alone (n = 3), single-cell RNA-seq (n = 1) and IHC combined with either an RNase protection assay or mass spectrometry (n = 2). B cells were detected in the majority (n = 13) by one of the commonly used B-cell markers alone (CD19, CD20 or CD22), a combination of B-cell markers, CD20 with either CD138 or HLA-DR and CD5 (n = 2) or CD138 and MUM (n = 1), or CD138 alone (n = 8). In addition, two studies examined the Ig expression (one study also included CD138), and only one single-cell RNA-seq study performed an unbiased profiling on endometrial B cells. It was also notable that no single-cell RNA-seq study has been performed on endometrial B cells in pathology-focused studies.

Despite the commonly reported claim that B cells are rare or absent in the endometrium (Lee *et al.*, 2011; Guzman-Genuino and Diener, 2017; Schumacher *et al.*, 2018; Ticconi *et al.*, 2019), the findings of this review show that endometrial B cells are consistently present in normal cycling human endometrium and endometrium from women with endometriosis, infertility, RIF, RPL, endometritis and other pathologies including abnormal uterine bleeding, endometrial polyps and uterine fibroids. B cells typically constitute <5% of total CD45^+^ lymphocytes, and no more than 2% of total cells in the normal endometrium. This is lower than in other lymphoid mucosal tissues such as the lung and gut (Farstad *et al.*, 2000; Weisel *et al.*, 2020), but is similar to tissues such as the stomach and liver. Gastric B cells account for 0–5% of all live cells in the antral mucosa biopsies from healthy stomach and hepatic B cells represent 1–8% of all lymphocytes in healthy liver (Norris *et al.*, 1998; Goll *et al.*, 2005; Robinson *et al.*, 2016). Although B cells are not an abundant cell population in these organs, their proportional and phenotypical differences as well as functional importance have been noted in gastric and hepatic diseases, such as peptic ulcer disease and hepatitis C infections (Racanelli *et al.*, 2001; Curry *et al.*, 2003; Goll *et al.*, 2005). By comparison, the characterization of endometrial B cells in reproductive pathologies is limited and has mainly focused on the proportional differences amongst tissues. A recent paper identified the presence of naïve B cells, memory B cells and plasma cells in ectopic endometrial tissue, eutopic endometrial tissue and normal endometrial tissues by analysing 309 cases of gene transcriptome data in endometriosis research. A positive correlation of naïve B cells with CD4 memory activated T cells (r^2^ = 0.7) and resting dendritic cells (r^2^ = 0.62), and a negative correlation of naïve B cells with memory B cells (r^2^ = −0.52), was observed in ectopic endometrial tissue (Zhong *et al.*, 2021). However, as the transcriptomic data were pulled from a public database, the patient characteristics were unclear (age, menstrual cycle phase when samples were obtained, etc.), which may have a direct impact on their immune cell composition.

In this systematic review, the results showed that the quantity of B cells within the endometrium is altered in certain pathologies. CD20^+^ B cells are more abundant in patients with endometritis, but data are conflicting as to whether there are increased B-cell numbers in patients with endometriosis, infertility, RIF and RPL. This is partially a result of the different methodologies used for B-cell identification: flow cytometric analysis involves tissue digestion, which might affect the surface expression of certain molecules and cause skewed cell population retrieval (Autengruber *et al.*, 2012; Skulska *et al.*, 2019); IHC only detects the immune profile of a limited tissue area, thus reliable quantification can be difficult to obtain. Discrepancies also arise between studies in the selection criteria for cases and controls, particularly in studies focused on RIF and RPL. Appropriate endometrial control tissue is often problematic in studies and this may be of relevance in the studies included in this review. Most studies used endometrium from women with proven fertility as controls. B cells present in the endometrium of these parous women may have acquired a distinct phenotype during pregnancy, therefore using fertile women as controls may reflect pregnancy-induced changes in B-cell populations rather than changes associated with the pathology of interest. Long-lived pregnancy-induced immune cell changes have been previously identified in endometrial NK cells where pregnancy trained decidual NK cells (PTdNKs) have been reported (Nielsen, 2011; Gamliel *et al.*, 2018). The definitions of RPL and RIF are still debated and differ between international guidelines (Rinehart, 2007; Youssef *et al.*, 2019). For example, the most recent ESHRE guideline defined RPL as the loss of two or more pregnancies (Bender Atik *et al.*, 2018), while most good quality studies presented in this systematic review defined RPL as the loss of three or more pregnancies. Similarly, for CE, there is no universally accepted standardized definition or established diagnostic guideline, although the identification of plasma cells (CD138 IHC staining) in the endometrial stroma is typically used. However, CD138 expression is not limited to plasma cells and cannot be considered a reliable marker alone; for example, CD138 is constitutively expressed on endometrial epithelial cells (Inki, 1997; Kind *et al.*, 2019; Moreno and Simon, 2019). Other plasma cell markers, such as MUM1, have also been explored (Parks *et al.*, 2018). Although IHC for MUM1 seems to have an improved sensitivity compared with CD138, MUM1 expression has been reported in 1–5% of tonsil and splenic T cells (largely GC T cells) as well as phytohemagglutinin activated T cells (Falini *et al.*, 2000, 1). We know T cells exhibit an activated phenotype in the human endometrium (Feyaerts *et al.*, 2017), therefore MUM1 expression alone might not be an ideal endometrial plasma cell marker and additional validation is required to ensure endometrial T cells do not express MUM1. Plasma cells do exhibit a distinct histological morphology, namely a round-to-ovoid shape and abundant cytoplasm, which might be useful as additional criteria in IHC identification (Allen and Sharma, 2020; Tellier and Nutt, 2017).

### Possible TLSs in endometrium

In total, 11 IHC studies investigated endometrial B cells using pan-B-cell markers such as CD19, CD20 or CD22. Eight of these studies described that B cells were present not only as single-cell entities but also in structures called LAs. Although the remaining papers did not report the presence of endometrial B cells in LAs, it is possible that a standard endometrial biopsy failed to aspirate the lower functional layer of the endometrium and/or the basal layer of the endometrium, where LAs are found (Fernández-Shaw *et al.*, 1995; Mettler *et al.*, 1997). LAs are predominantly found with a B-cell core, surrounded by T cells and macrophages (Fig. 2). These B cells have been documented in low but constant numbers throughout the menstrual cycle; however, their phenotype and function are relatively unexplored (Mettler *et al.*, 1997; Wira *et al.*, 2014).

LAs resemble ectopic/ TLSs that have been described in various tissues in response to chronic immune stimulation. Their function can be both protective (in cancers and tumours) and destructive (largely in autoimmune conditions) (Pipi *et al.*, 2018). TLS formation and structure are unique to the tissue in which they arise, however, they may be generally described as organized B-cell compartments containing GCs, with T-cell compartments housing antigen-presenting cells, and in many tissues, high endothelial venules allowing entry of lymphocytes from the peripheral circulation (van de Pavert and Mebius, 2010). In comparison, LAs are composed of a B-cell core and a surrounding spheroidal predominantly T-cell mass, with a diffuse halo of macrophages (Mettler *et al.*, 1997; Wira *et al.*, 2014). There is debate in the literature as to the defining characteristics of TLSs; however, LAs may constitute a novel TLS, albeit after adopting a more inclusive definition than that which has been historically proposed (Table VI). Further evidence is needed to confirm the cellular phenotype of LAs, structure and functional properties; for example, there has been little exploration into whether these B cells may constitute GC-like B cells. Studies investigating the presence of GC-like structures and associated cells have failed to use specific GC markers to support their findings (Morris *et al.*, 1985; Wira *et al.*, 2014).

**Table VI:** Comparison of the bona fide definition of tertiary lymphoid structures with evidence from human endometrial lymphoid aggregates.

**Bona fide definition of tertiary lymphoid structures** (Dieu-Nosjean *et al*., 2014)	Evidence from endometrial lymphoid aggregates	References
Ectopic and organized lymphoid tissue, contains distinct T- and B-cell zones	Yes	Disep *et al.*, (2004); Klentzeris *et al.* (1992); Mettler *et al.* (1997); Wira *et al.* (2014)
PNAd^+^ high endothelial venules present surrounding, for extravasation of CD26L^+^ immune cells	Addressins of the PNAd family have not been examined for endometrial LAs; however, other adhesion molecules including ICAM-1, VCAM-1 and E-selectin have been reported. Lymphoid aggregates with CD20^+^ B cell have been detected in the surroundings of MECA-79^+^ high endothelial venules.	Tabibzadeh *et al*., (1994); Windsperger *et al*., (2020)
Evidence of B-cell class switching and GC reactions in B-cell follicles	GC B cells (FAS^+^ IgD^−^ BCL6^+^) have been identified in human endometrium, but the functional activity has not been studied.	Shen *et al.* (2021)
LAMP^+^ mature dendritic cells present in T-cell zone	LAMP^+^ dendritic cells have been reported in the endometrium; however, they have not been explicitly examined in LAs. Dendritic reticulum cells (DRC) have been noted in the LAs, with staining conducted using anti-DRC1 antibody.	Maridas *et al*., (2014); Schulke *et al*., (2008)
Expression of major set of chemokines involved in SLO organization	Chemokine expression has not been studied.	–

DRC1, dendritic reticulum cells; GC, germinal centre; ICAM-1, intracellular adhesion molecule; LAMP, lysosome associated membrane protein; LAMP, lysosome-associated membrane protein; MECA-79, high endothelial venule marker monoclonal antibody; PNAd, peripheral node addressin; VCAM-1, vascular cell adhesion molecule.

The mechanism by which LAs grow across the menstrual cycle is not entirely understood, but it is possible that the aggregate structure is under hormonal control (Yeaman *et al.*, 1997, 2001). In general, LAs express HLA-DR (Mettler *et al.*, 1997), suggesting they could play an active role in immune protection during menstruation, possibly through antigen presenting in a GC-like structure. This GC-like structure may contribute to the generation of class-switched B cells and plasmablasts found in the decidua (Huang *et al.*, 2017; Leng *et al.*, 2019). Additionally, the infiltration of plasma cells has been described in endometrial inflammatory conditions such as endometritis. It is possible that LA GC-like B cells are implicated in this process by providing the site of plasma cell generation. However, visualization of GC-like B cells and associated cells necessary for propagating their activation, including T-follicular helper cells, is needed to confirm this hypothesis. Visualization of these structures could further classify LAs as a TLS, which may assist in their characterization by identifying common pathways and molecules documented as being involved with various TLS formation and maintenance processes.

### Conclusions and clinical implications

Evidence from our systematic review has demonstrated that endometrial B cells are persistently present in normal human endometrium, accounting for no more than 5% of all CD45^+^ lymphocytes. Their relative abundance and phenotypes vary in benign gynaecological conditions, but the current evidence is inconsistent. Endometrial B cells are either scattered or surrounded by T cells and macrophages in LAs. LAs seem to be under hormonal control throughout the menstrual cycle and are similar to TLSs that have been observed in various tissues.

This work synthesizes the current evidence about our understanding of endometrial B cells and identifies future research questions. Firstly, endometrial B-cell subsets and their expressed tissue residency markers need to be determined, ideally through unbiased profiling, such as RNA sequencing, or combining flow cytometry and IHC. Single-cell sequencing would also significantly advance our understanding of B cells but for minority cell populations such as endometrial B cells, cell enrichment is a necessary step before sequencing in order to obtain enough data to define B-cell sub-populations. Our group recently performed flow cytometric and transcriptomic (RNA-seq) analysis on endometrial B cells, illustrating that most mid-luteal endometrial B cells are IgM^+^ CD27^−^ naïve and CD27^+^ memory B cells. A GC-like B-cell (FAS^+^ IgD^−^ BCL6^+^) population was also identified, representing 12.2 ± 8.6% of all endometrial B cells. Previous studies demonstrated that endometrial B-cell lymphoma-6 (BCL6) protein levels are significantly higher in patients with RPL, unexplained infertility or endometriosis (eutopic endometrium) when compared with controls (Evans-Hoeker *et al.*, 2016; Almquist *et al.*, 2017; Fox *et al.*, 2020; Sansone *et al.*, 2021). However, BCL6 expression is not limited to endometrial B cells, therefore, it is not yet clear whether the up-regulation of BCL6 is a reflection of the existence and/or involvement of GC-like B cells in the endometrial environment. Additionally, endometrial B cells exhibited an MHC class II activated phenotype, with increased expression levels of CD74, CD83 and HLA-DR compared with circulating B cells, suggesting an antigen-presenting function of endometrial B cells (Shen *et al.*, 2021). Although our research expanded the current understanding of endometrial B cells, our sample group contains a mixture of controls and patients with RPL and RIF. Further research is needed to investigate whether there are any phenotypic or functional alterations to endometrial B cells in reproductive pathologies. Secondly, the functional properties and significance of endometrial LAs need additional investigation, including the identification and spatial visualization of the component cells, focusing on TLS related T follicular helper and GC B cells. Utilizing spatial profiling techniques to map cell proteins between loose stromal cells and cells of the LA would help us understand the cellular component and function of endometrial LAs. Finally, the reliability of diagnosing endometritis using CD138 alone to define a plasma cell warrants further study.

Addressing these questions would be important to advance our understanding of the role of B cells in the normal endometrium and benign endometrial pathologies, especially in autoimmune associated reproductive pathologies, such as endometriosis, where an early study in mice demonstrated that B-cell inactivation induced by a Bruton’s tyrosine kinase (Btk) inhibitor (ibrutinib) limits endometriosis progression (Riccio *et al.*, 2019). For CE, an advanced understanding of endometrial B cells and plasma cells would help to standardize its diagnosis and management in clinical practice.

## Supplementary data

Supplementary data are available at *Human Reproduction Open* online.

## Data availability

No new data were generated or analysed in support of this research.

## Authors’ roles

M.S., P.M., J.S. and I.G. conceived of the study. Data were generated and interpreted by M.S., E.O’.D. and A.K. All authors contributed to refinement of the study protocol and approved the final manuscript.

## Funding

This study was funded by Finox Biotech. M.S. received a PhD grant from The British Federation of Women Graduates (BFWG) and Sir Richard Stapley Educational Trust.

## Conflict of interest

The authors have no conflicts of interest to declare.

## Supplementary Material

hoab043_Supplementary_Data

## References

[hoab043-B1] Adachi Y , Onodera T, Yamada Y, Daio R, Tsuiji M, Inoue T, Kobayashi K, Kurosaki T, Ato M, Takahashi Y. Distinct germinal center selection at local sites shapes memory B cell response to viral escape. J Exp Med 2015;212:1709–1723. 26324444 10.1084/jem.20142284 PMC4577849

[hoab043-B2] Allen HC , Sharma P. Histology plasma cells. In: StatPearls. Treasure Island (FL): StatPearls Publishing, 2020. http://www.ncbi.nlm.nih.gov/books/NBK556082/ (1 October 2021, date last accessed). 32310542

[hoab043-B3] Almquist LD , Likes CE, Stone B, Brown KR, Savaris R, Forstein DA, Miller PB, Lessey BA. Endometrial BCL6 testing for the prediction of in vitro fertilization outcomes: a cohort study. Fertil Steril 2017;108:1063–1069. 29126613 10.1016/j.fertnstert.2017.09.017 PMC5726554

[hoab043-B93] American Fertility Society Classification of Endometriosis: 1985. Fertil Steril 1985;43:351–352. 3979573 10.1016/s0015-0282(16)48430-x

[hoab043-B4] Antsiferova YS , Sotnikova NYu, Posiseeva LV, Shor AL. Changes in the T-helper cytokine profile and in lymphocyte activation at the systemic and local levels in women with endometriosis. Fertil Steril 2005;84:1705–1711. 16359969 10.1016/j.fertnstert.2005.05.066

[hoab043-B5] Autengruber A , Gereke M, Hansen G, Hennig C, Bruder D. Impact of enzymatic tissue disintegration on the level of surface molecule expression and immune cell function. Eur J Microbiol Immunol 2012;2:112–120. 10.1556/EuJMI.2.2012.2.3 PMC3956959 24672679

[hoab043-B6] Bender Atik R , Christiansen OB, Elson J, Kolte AM, Lewis S, Middeldorp S, Nelen W, Peramo B, Quenby S, Vermeulen N et al ESHRE guideline: recurrent pregnancy loss. Hum Reprod Open 2018;2018:hoy004. 10.1093/hropen/hoy004 PMC6276652 31486805

[hoab043-B7] Bohlmann MK , Luedders DW, Strowitzki T, von Wolff M. Specific secretory phase endometrial leukocytes of women with two and more consecutive idiopathic abortions are not significantly different from healthy controls. Arch Gynecol Obstet 2010;281:983–990. 19847451 10.1007/s00404-009-1179-9

[hoab043-B8] Brandtzaeg P. Induction of secretory immunity and memory at mucosal surfaces. Vaccine 2007;25:5467–5484. 17227687 10.1016/j.vaccine.2006.12.001

[hoab043-B9] Burgener A , Tjernlund A, Kaldensjo T, Abou M, McCorrister S, Westmacott GR, Mogk K, Ambrose E, Broliden K, Ball B. A systems biology examination of the human female genital tract shows compartmentalization of immune factor expression. J Virol 2013;87:5141–5150. 23449785 10.1128/JVI.03347-12 PMC3624310

[hoab043-B10] Chen C-K , Huang S-C, Chen C-L, Yen M-R, Hsu H-C, Ho H-N. Increased expressions of CD69 and HLA-DR but not of CD25 or CD71 on endometrial T lymphocytes of nonpregnant women. Hum Immunol 1995;42:227–232. 7759310 10.1016/0198-8859(94)00105-y

[hoab043-B11] Cicinelli E , Bettocchi S, de Ziegler D, Loizzi V, Cormio G, Marinaccio M, Trojano G, Crupano FM, Francescato R, Vitagliano A et al Chronic endometritis, a common disease hidden behind endometrial polyps in premenopausal women: first evidence from a case-control study. J Minim Invasive Gynecol 2019;26:1346–1350. 30708117 10.1016/j.jmig.2019.01.012

[hoab043-B12] Curry MP , Golden ML, Doherty DG, Deignan T, Norris S, Duffy M, Nolan N, Hall W, Hegarty JE, O’Farrelly C. Expansion of innate CD5pos B cells expressing high levels of CD81 in hepatitis C virus infected liver. J Hepatol 2003;38:642–650. 12713876 10.1016/s0168-8278(03)00075-8

[hoab043-B13] Danaii S , Ghorbani F, Ahmadi M, Abbaszadeh H, Koushaeian L, Soltani-Zangbar MS, Mehdizadeh A, Hojjat-Farsangi M, Kafil HS, Aghebati-Maleki L et al IL-10-producing B cells play important role in the pathogenesis of recurrent pregnancy loss. Int Immunopharmacol 2020;87:106806. 32693358 10.1016/j.intimp.2020.106806

[hoab043-B46028699] Dieu-Nosjean M-C , Goc J, Giraldo NA, Sautès-Fridman C, Fridman WH. Tertiary lymphoid structures in cancer and beyond. *Trends Immunol* 2014;35:571–580. 25443495 10.1016/j.it.2014.09.006

[hoab043-B14] Disep B , Innes BA, Cochrane HR, Tijani S, Bulmer JN. Immunohistochemical characterization of endometrial leucocytes in endometritis. Histopathology 2004;45:625–632. 15569054 10.1111/j.1365-2559.2004.02052.x

[hoab043-B15] Esteve-Solé A , Luo Y, Vlagea A, Deyà-Martínez Á, Yagüe J, Plaza-Martín AM, Juan M, Alsina L. B regulatory cells: players in pregnancy and early life. Int J Mol Sci 2018;19:2099–2116. 10.3390/ijms19072099 PMC6073150 30029515

[hoab043-B16] Evans-Hoeker E , Lessey BA, Jeong JW, Savaris RF, Palomino WA, Yuan L, Schammel DP, Young SL. Endometrial BCL6 overexpression in eutopic endometrium of women with endometriosis. Reprod Sci 2016;23:1234–1241. 27222232 10.1177/1933719116649711 PMC5933165

[hoab043-B17] Falini B , Fizzotti M, Pucciarini A, Bigerna B, Marafioti T, Gambacorta M, Pacini R, Alunni C, Natali–Tanci L, Ugolini B et al A monoclonal antibody (MUM1p) detects expression of the MUM1/IRF4 protein in a subset of germinal center B cells, plasma cells, and activated T cells. Blood 2000;95:2084–2092. 10706878

[hoab043-B18] Fan X , Yang Y, Wen Q, Li Y, Meng F, Liao J, Chen H, Lu G-X, Lin G, Gong F. CD19 and intraglandular CD163-positivity as prognostic indicators of pregnancy outcome in CD138-negative women with a previous fresh-embryo-transfer failure. J Reprod Immunol 2021;147:103362. 34482188 10.1016/j.jri.2021.103362

[hoab043-B19] Farstad IN , Carlsen H, Morton HC, Brandtzaeg P. Immunoglobulin A cell distribution in the human small intestine: phenotypic and functional characteristics. Immunology 2000;101:354–363. 11106939 10.1046/j.1365-2567.2000.00118.x PMC2327091

[hoab043-B20] Fernández-Shaw S , Clarke MT, Hicks B, Naish CE, Barlow DH, Starkey PM. Bone marrow-derived cell populations in uterine and ectopic endometrium. Hum Reprod 1995;10:2285–2289. 8530653 10.1093/oxfordjournals.humrep.a136286

[hoab043-B21] Feyaerts D , Benner M, van Cranenbroek B, van der Heijden OWH, Joosten I, van der Molen RG. Human uterine lymphocytes acquire a more experienced and tolerogenic phenotype during pregnancy. Sci Rep 2017;7:2884–2894. 28588205 10.1038/s41598-017-03191-0 PMC5460245

[hoab043-B22] Fox CW , Savaris RF, Jeong J-W, Kim TH, Miller PB, Likes CE, Schammel DP, Young SL, Lessey BA. Unexplained recurrent pregnancy loss and unexplained infertility: twins in disguise. Hum Reprod Open 2020;2020:hoz021. 10.1093/hropen/hoz021 PMC9869655 36694811

[hoab043-B23] Gagné D , Rivard M, Pagé M, Shazand K, Hugo P, Gosselin D. Blood leukocyte subsets are modulated in patients with endometriosis. Fertil Steril 2003;80:43–53. 12849800 10.1016/s0015-0282(03)00552-1

[hoab043-B24] Gamliel M , Goldman-Wohl D, Isaacson B, Gur C, Stein N, Yamin R, Berger M, Grunewald M, Keshet E, Rais Y et al Trained memory of human uterine NK cells enhances their function in subsequent pregnancies. Immunity 2018;48:951–962.e5. 29768178 10.1016/j.immuni.2018.03.030

[hoab043-B25] Goll R , Husebekk A, Isaksen V, Kauric G, Hansen T, Florholmen J. Increased frequency of antral CD4+ T and CD19+ B cells in patients with helicobacter pylori-related peptic ulcer disease. Scand J Immunol 2005;61:92–97. 15644128 10.1111/j.0300-9475.2005.01537.x

[hoab043-B26] Guzman-Genuino RM , Diener KR. Regulatory B cells in pregnancy: lessons from autoimmunity, graft tolerance, and cancer. Front Immunol 2017;8:1–13. 28261223 10.3389/fimmu.2017.00172 PMC5313489

[hoab043-B27] Hershberg U , Luning Prak ET. The analysis of clonal expansions in normal and autoimmune B cell repertoires. Phil Trans R Soc B 2015;370:20140239. 26194753 10.1098/rstb.2014.0239 PMC4528416

[hoab043-B28] Holzer I , Ott J, Kurz C, Hofstetter G, Hager M, Kuessel L, Parry JP. Is chronic endometritis associated with tubal infertility? A prospective cohort study. J Minim Invasive Gynecol 2021;28:1876–1881. 33892185 10.1016/j.jmig.2021.04.011

[hoab043-B29] Huang B , Faucette AN, Pawlitz MD, Pei B, Goyert JW, Zhou JZ, El-Hage NG, Deng J, Lin J, Yao F et al Interleukin-33-induced expression of PIBF1 by decidual B cells protects against preterm labor. Nat Med 2017;23:128–135. 27918564 10.1038/nm.4244 PMC5512431

[hoab043-B30] Huang W , Liu B, He Y, Xie Y, Liang T, Bi Y, Yuan L, Qin A, Wang Y, Yang Y. Variation of diagnostic criteria in women with chronic endometritis and its effect on reproductive outcomes: a systematic review and meta-analysis. J Reprod Immunol 2020;140:103146. 32442825 10.1016/j.jri.2020.103146

[hoab043-B31] Inki P. Expression of syndecan-1 in female reproductive tract tissues and cultured keratinocytes. Mol Hum Reprod 1997;3:299–305. 9237257 10.1093/molehr/3.4.299

[hoab043-B89863931] Johnson NP , Hummelshoj L, Adamson GD, Keckstein J, Taylor HS, Abrao MS, Bush D, Kiesel L, Tamimi R, Sharpe-Timms KL et al World Endometriosis Society consensus on the classification of endometriosis. *Hum Reprod* 2017;32:315–324. 27920089 10.1093/humrep/dew293

[hoab043-B32] Khizroeva J , Nalli C, Bitsadze V, Lojacono A, Zatti S, Andreoli L, Tincani A, Shoenfeld Y, Makatsariya A. Infertility in women with systemic autoimmune diseases. Best Pract Res Clin Endocrinol Metab 2019;33:101369. 31837981 10.1016/j.beem.2019.101369

[hoab043-B33] Kind S , Merenkow C, Büscheck F, Möller K, Dum D, Chirico V, Luebke AM, Höflmayer D, Hinsch A, Jacobsen F et al Prevalence of syndecan-1 (CD138) expression in different kinds of human tumors and normal tissues. Dis Markers 2019;2019:4928315. 31976021 10.1155/2019/4928315 PMC6954471

[hoab043-B34] King A , Burrows T, Verma S, Hiby S, Loke YW. Human uterine lymphocytes. Hum Reprod Update 1998;4:480–485. 10027599 10.1093/humupd/4.5.480

[hoab043-B35] Kitaya K , Tada Y, Hayashi T, Taguchi S, Funabiki M, Nakamura Y. Comprehensive endometrial immunoglobulin subclass analysis in infertile women suffering from repeated implantation failure with or without chronic endometritis. Am J Reprod Immunol 2014;72:386–391. 24898900 10.1111/aji.12277

[hoab043-B36] Kitaya K , Yasuo T. Aberrant expression of selectin E, CXCL1, and CXCL13 in chronic endometritis. Mod Pathol2010a;23:1136–1146. 20495539 10.1038/modpathol.2010.98

[hoab043-B37] Kitaya K , Yasuo T. Leukocyte density and composition in human cycling endometrium with uterine fibroids. Hum Immunol2010b;71:158–163. 19961890 10.1016/j.humimm.2009.11.014

[hoab043-B38] Klentzeris LD , Bulmer JN, Liu DT, Morrison L. Endometrial leukocyte subpopulations in women with endometriosis. Eur J Obstetr Gynecol Reprod Biol 1995;63:41–47. 10.1016/0301-2115(95)02222-s 8674564

[hoab043-B39] Klentzeris LD , Bulmer JN, Warren A, Morrison L, Li TC, Cooke ID. Endometrial lymphoid tissue in the timed endometrial biopsy: morphometric and immunohistochemical aspects. Am J Obstet Gynecol 1992;167:667–674. 1530020 10.1016/s0002-9378(11)91568-3

[hoab043-B40] Klentzeris LD , Bulmer JN, Warren MA, Morrison L, Li TC, Cooke ID. Lymphoid tissue in the endometrium of women with unexplained infertility: morphometric and immunohistochemical aspects. Hum Reprod 1994;9:646–652. 7519197 10.1093/oxfordjournals.humrep.a138564

[hoab043-B41] Koboziev I , Karlsson F, Grisham MB. Gut-associated lymphoid tissue, T cell trafficking, and chronic intestinal inflammation. Ann N Y Acad Sci 2010;1207:E86–E93. 20961311 10.1111/j.1749-6632.2010.05711.x PMC3075575

[hoab043-B42] Lachapelle MH , Miron P, Hemmings R, Roy DC. Endometrial T, B, and NK cells in patients with recurrent spontaneous abortion. Altered profile and pregnancy outcome. J Immunol 1996;156:4027–4034. 8621945

[hoab043-B43] LeBien TW , Tedder TF. B lymphocytes: how they develop and function. Blood 2008;112:1570–1580. 18725575 10.1182/blood-2008-02-078071 PMC2518873

[hoab043-B44] Lee JY , Lee M, Lee SK. Role of endometrial immune cells in implantation. Clin Exp Reprod Med 2011;38:119–125. 22384430 10.5653/cerm.2011.38.3.119 PMC3283071

[hoab043-B45] Leng Y , Romero R, Xu Y, Galaz J, Slutsky R, Arenas-Hernandez M, Garcia-Flores V, Motomura K, Hassan SS, Reboldi A et al Are B cells altered in the decidua of women with preterm or term labor? Am J Reprod Immunol 2019;81:e13102. 30768818 10.1111/aji.13102 PMC6556388

[hoab043-B46] Li Y , Xu S, Yu S, Huang C, Lin S, Chen W, Mo M, Lian R, Diao L, Ding L et al Diagnosis of chronic endometritis: how many CD138+ cells/HPF in endometrial stroma affect pregnancy outcome of infertile women? Am J Reprod Immunol 2021;85:e13369. 33152123 10.1111/aji.13369

[hoab043-B47] Liu Y , Chen X, Huang J, Wang C-C, Yu M-Y, Laird S, Li T-C. Comparison of the prevalence of chronic endometritis as determined by means of different diagnostic methods in women with and without reproductive failure. Fertil Steril 2018;109:832–839. 29778382 10.1016/j.fertnstert.2018.01.022

[hoab043-B48] Lucas ES , Vrljicak P, Muter J, Diniz-da-Costa MM, Brighton PJ, Kong C-S, Lipecki J, Fishwick KJ, Odendaal J, Ewington LJ et al Recurrent pregnancy loss is associated with a pro-senescent decidual response during the peri-implantation window. Commun Biol 2020;3:14. 31965050 10.1038/s42003-020-0763-1 PMC6972755

[hoab043-B8724197] Maridas DE , Hey-Cunningham AJ, Ng CHM, Markham R, Fraser IS, Berbic M. Peripheral and endometrial dendritic cell populations during the normal cycle and in the presence of endometriosis. *J Endometr Pelvic Pain Disord* 2014;6:67–119. 29354600 10.5301/je.5000180 PMC5771262

[hoab043-B49] McGhee JR , Fujihashi K. Inside the mucosal immune system. PLoS Biol 2012;10:e1001397. 23049482 10.1371/journal.pbio.1001397 PMC3457930

[hoab043-B50] McQueen DB , Maniar KP, Hutchinson A, Confino R, Bernardi L, Pavone ME. Redefining chronic endometritis: the importance of endometrial stromal changes. Fertil Steril 2021;116:855–861. 34120737 10.1016/j.fertnstert.2021.04.036

[hoab043-B51] Mettler L , Jürgensen A, Volkov NI, Kulakov V, Parwaresch MR. lmmuno histochemical profile of endometrium in patients with genital endometriosis. Diagn Ther Endosc 1997;3:127–145. 18493428 10.1155/DTE.3.127 PMC2362564

[hoab043-B52] Michimata T , Ogasawara MS, Tsuda H, Suzumori K, Aoki K, Sakai M, Fujimura M, Nagata K, Nakamura M, Saito S. Distributions of endometrial NK cells, B cells, T cells, and Th2/Tc2 cells fail to predict pregnancy outcome following recurrent abortion. Am J Reprod Immunol 2002;47:196–202. 12069386 10.1034/j.1600-0897.2002.01048.x

[hoab043-B53] Modesti PA , Reboldi G, Cappuccio FP, Agyemang C, Remuzzi G, Rapi S, Perruolo E, Parati G; ESH Working Group on CV Risk in Low Resource Settings. Panethnic differences in blood pressure in Europe: a systematic review and meta-analysis. PLoS One 2016;11:e0147601. 26808317 10.1371/journal.pone.0147601 PMC4725677

[hoab043-B54] Moreno I , Simon C. An endometrial pathology in the inflammation cloud that can be accessed with a microbial app. Fertil Steril 2019;111:679–680. 30871763 10.1016/j.fertnstert.2018.12.022

[hoab043-B55] Morris H , Edwards J, Tiltman A, Emms M. Endometrial lymphoid tissue: an immunohistological study. J Clin Pathol 1985;38:644–652. 3891790 10.1136/jcp.38.6.644 PMC499262

[hoab043-B56] Muzzio D , Zenclussen AC, Jensen F. The role of B cells in pregnancy: the good and the bad. Am J Reprod Immunol 2013;69:408–412. 23351028 10.1111/aji.12079

[hoab043-B57] Nielsen HS. Secondary recurrent miscarriage and H-Y immunity. Hum Reprod Update 2011;17:558–574. 21482560 10.1093/humupd/dmr005

[hoab043-B58] Norris S , Collins C, Doherty DG, Smith F, McEntee G, Traynor O, Nolan N, Hegarty J, O'Farrelly C. Resident human hepatitis lymphocytes are phenotypically different from circulating lymphocytes. J Hepatol 1998;28:84–90. 9537869 10.1016/s0168-8278(98)80206-7

[hoab043-B59] O’Donnell E , Shen M, Becker C, Lindgren C, Zondervan K, Southcombe J, Granne I, Melo P. The role of human endometrial B cells in health and benign female reproductive pathologies: a systematic review. *PROSPERO 2020 CRD42020152915* 2020. https://www.crd.york.ac.uk/prospero/display_record.php?ID=CRD42020152915 (1 October 2021, date last accessed).

[hoab043-B60] Page MJ , McKenzie JE, Bossuyt PM, Boutron I, Hoffmann TC, Mulrow CD, Shamseer L, Tetzlaff JM, Akl EA, Brennan SE et al The PRISMA 2020 statement: an updated guideline for reporting systematic reviews. BMJ 2021;372:n71. 33782057 10.1136/bmj.n71 PMC8005924

[hoab043-B61] Parks RN , Kim CJ, Al-Safi ZA, Armstrong AA, Zore T, Moatamed NA. Multiple myeloma 1 transcription factor is superior to CD138 as a marker of plasma cells in endometrium. Int J Surg Pathol 2018;27:372–379. 30482071 10.1177/1066896918814307

[hoab043-B62] Pipi E , Nayar S, Gardner DH, Colafrancesco S, Smith C, Barone F. Tertiary lymphoid structures: autoimmunity goes local. Front Immunol 2018;9:1–21. 30258435 10.3389/fimmu.2018.01952 PMC6143705

[hoab043-B63] Quenby S , Bates M, Doig T, Brewster J, Lewis-Jones DI, Johnson PM, Vince G. Pre-implantation endometrial leukocytes in women with recurrent miscarriage. Hum Reprod 1999;14:2386–2391. 10469717 10.1093/humrep/14.9.2386

[hoab043-B64] Racanelli V , Sansonno D, Piccoli C, D'Amore FP, Tucci FA, Dammacco F. Molecular characterization of B cell clonal expansions in the liver of chronically hepatitis C virus-infected patients. J Immunol 2001;167:21–29. 11418627 10.4049/jimmunol.167.1.21

[hoab043-B65] Rawlings DJ , Metzler G, Wray-Dutra M, Jackson SW. Altered B cell signalling in autoimmunity. Nat Rev Immunol 2017;17:421–436. 28393923 10.1038/nri.2017.24 PMC5523822

[hoab043-B66] Riccio LGC , Baracat EC, Chapron C, Batteux F, Abrão MS. The role of the B lymphocytes in endometriosis: a systematic review. J Reprod Immunol 2017;123:29–34. 28910679 10.1016/j.jri.2017.09.001

[hoab043-B67] Riccio LGC , Jeljeli M, Santulli P, Chouzenoux S, Doridot L, Nicco C, Reis FM, Abrão MS, Chapron C, Batteux F. B lymphocytes inactivation by Ibrutinib limits endometriosis progression in mice. Hum Reprod 2019;34:1225–1234. 31247078 10.1093/humrep/dez071

[hoab043-B68] Rinehart J. Recurrent implantation failure: definition. J Assist Reprod Genet 2007;24:284–287. 17674185 10.1007/s10815-007-9147-4 PMC3455006

[hoab043-B69] Robinson MW , Harmon C, O'Farrelly C. Liver immunology and its role in inflammation and homeostasis. Cell Mol Immunol 2016;13:267–276. 27063467 10.1038/cmi.2016.3 PMC4856809

[hoab043-B70] Ruddle NH , Akirav EM. Secondary lymphoid organs: responding to genetic and environmental cues in ontogeny and the immune response. J Immunol 2009;183:2205–2212. 19661265 10.4049/jimmunol.0804324 PMC2766168

[hoab043-B71] Sansone AM , Hisrich BV, Young RB, Abel WF, Bowens Z, Blair BB, Funkhouser AT, Schammel DP, Green LJ, Lessey BA et al Evaluation of BCL6 and SIRT1 as non-invasive diagnostic markers of endometriosis. Curr Issues Mol Biol 2021;43:1350–1360. 34698105 10.3390/cimb43030096 PMC8929102

[hoab043-B5958993] Schulke L , Manconi F, Markham R, Fraser IS. Endometrial dendritic cell populations during the normal menstrual cycle. *Human Reproduction* 2008;23:1574–1580. 18285323 10.1093/humrep/den030

[hoab043-B72] Schumacher A , Sharkey DJ, Robertson SA, Zenclussen AC. Immune cells at the fetomaternal interface: how the microenvironment modulates immune cells to foster fetal development. J Immunol 2018;201:325–334. 29987001 10.4049/jimmunol.1800058

[hoab043-B73] Shen M , Child T, Mittal M, Sarodey G, Salim R, Granne I, Southcombe JH. B cell subset analysis and gene expression characterization in mid-luteal endometrium. Front Cell Dev Biol 2021;9:709280. 34447753 10.3389/fcell.2021.709280 PMC8383145

[hoab043-B74] Shigesi N , Kvaskoff M, Kirtley S, Feng Q, Fang H, Knight JC, Missmer SA, Rahmioglu N, Zondervan KT, Becker CM. The association between endometriosis and autoimmune diseases: a systematic review and meta-analysis. Hum Reprod Update 2019;25:486–503. 31260048 10.1093/humupd/dmz014 PMC6601386

[hoab043-B75] Skulska K , Wegrzyn AS, Chelmonska-Soyta A, Chodaczek G. Impact of tissue enzymatic digestion on analysis of immune cells in mouse reproductive mucosa with a focus on γδ T cells. J Immunol Methods 2019;474:112665. 31525366 10.1016/j.jim.2019.112665

[hoab043-B76] Song D , Li T-C, Zhang Y, Feng X, Xia E, Huang X, Xiao Y. Correlation between hysteroscopy findings and chronic endometritis. Fertil Steril 2019;111:772–779. 30683588 10.1016/j.fertnstert.2018.12.007

[hoab043-B055735] Tabibzadeh S , Kong QF, Babaknia A. Expression of adhesion molecules in human endometrial vasculature throughout the menstrual cycle. *J Clin Endocrinol Metab* 1994;79:1024–1032. 7962270 10.1210/jcem.79.4.7962270

[hoab043-B77] Tellier J , Nutt SL. Standing out from the crowd: How to identify plasma cells. Eur J Immunol 2017;47:1276–1279. 28787106 10.1002/eji.201747168

[hoab043-B78] Ticconi C , Pietropolli A, Di Simone N, Piccione E, Fazleabas A. Endometrial immune dysfunction in recurrent pregnancy loss. IJMS 2019;20:5332. 10.3390/ijms20215332 PMC6862690 31717776

[hoab043-B79] Toth M , Patton DL, Esquenazi B, Shevchuk M, Thaler H, Divon M. Association between *Chlamydia trachomatis* and abnormal uterine bleeding. Am J Reprod Immunol 2007;57:361–366. 17430500 10.1111/j.1600-0897.2007.00481.x

[hoab043-B80] Vajdy M. Generation and maintenance of mucosal memory B cell responses? Curr Med Chem 2006;13:3023–3037. 17073644 10.2174/092986706778521760

[hoab043-B81] Vallvé-Juanico J , Houshdaran S, Giudice LC. The endometrial immune environment of women with endometriosis. Hum Reprod Update 2019;25:565–592. 10.1093/humupd/dmz018 PMC6737540 31424502

[hoab043-B82] van de Pavert SA , Mebius RE. New insights into the development of lymphoid tissues. Nat Rev Immunol 2010;10:664–674. 20706277 10.1038/nri2832

[hoab043-B83] Weisel NM , Weisel FJ, Farber DL, Borghesi L, Shen Y, Ma W, Luning Prak ET, Shlomchik M. Comprehensive analyses of B cell compartments across the human body reveal novel subsets and a gut resident memory phenotype. Blood 2020;136:2774–2785. 32750113 10.1182/blood.2019002782 PMC7731793

[hoab043-B84] Wells G , Shea B, O’Connell D, Peterson J, Welch V, Losos M, Tugwell P. The Newcastle-Ottawa Scale (NOS) for assessing the quality of nonrandomised studies in meta-analyses. 2020. http://www.ohri.ca/programs/clinical_epidemiology/oxford.asp (1 October 2021, date last accessed).

[hoab043-B43692176] Windsperger K , Vondra S, Lackner AI, Kunihs V, Haslinger P, Meinhardt G, Dietrich B, Dekan S, Fiala C, Knöfler M et al Densities of decidual high endothelial venules correlate with T-cell influx in healthy pregnancies and idiopathic recurrent pregnancy losses. *Human Reproduction* 2020;35:2467–2477. 32940686 10.1093/humrep/deaa234

[hoab043-B85] Wira CR , Fahey JV, Rodriguez-Garcia M, Shen Z, Patel MV. Regulation of mucosal immunity in the female reproductive tract: the role of sex hormones in immune protection against sexually transmitted pathogens. Am J Reprod Immunol 2014;72:236–258. 24734774 10.1111/aji.12252 PMC4351777

[hoab043-B86] Yeaman GR , Collins JE, Fanger MW, Wira CR, Lydyard PM. CD8+ T cells in human uterine endometrial lymphoid aggregates: evidence for accumulation of cells by trafficking. Immunology 2001;102:434–440. 11328377 10.1046/j.1365-2567.2001.01199.x PMC1783206

[hoab043-B87] Yeaman GR , Guyre PM, Fanger MW, Collins JE, White HD, Rathbun W, Orndorff KA, Gonzalez J, Stern JE, Wira CR. Unique CD8+ T cell-rich lymphoid aggregates in human uterine endometrium. J Leukoc Biol 1997;61:427–435. 9103229

[hoab043-B88] Youssef A , Vermeulen N, Lashley EELO, Goddijn M, van der Hoorn MLP. Comparison and appraisal of (inter)national recurrent pregnancy loss guidelines. Reprod Biomed Online 2019;39:497–503. 31182358 10.1016/j.rbmo.2019.04.008

[hoab043-B89] Zargar M , Ghafourian M, Nikbakht R, Mir Hosseini V, Moradi Choghakabodi P. Evaluating chronic endometritis in women with recurrent implantation failure and recurrent pregnancy loss by hysteroscopy and immunohistochemistry. J Minim Invasive Gynecol 2020;27:116–121. 30851430 10.1016/j.jmig.2019.02.016

[hoab043-B90] Zhong Q , Yang F, Chen X, Li J, Zhong C, Chen S. Patterns of immune infiltration in endometriosis and their relationship to r-AFS stages. Front Genet 2021;12:631715. 34220927 10.3389/fgene.2021.631715 PMC8249861

[hoab043-B91] Zhou JZ , Way SS, Chen K. Immunology of the uterine and vaginal mucosae. Trends Immunol 2018;39:302–314. 29530651 10.1016/j.it.2018.02.006 PMC5880711

[hoab043-B92] Zondervan KT , Becker CM, Missmer SA. Endometriosis. N Engl J Med 2020;382:1244–1256. 32212520 10.1056/NEJMra1810764

