# Supplementary material for: The role of endometrial B cells in normal endometrium and benign female reproductive pathologies: a systematic review
Source: Hum Reprod Open. 2021 Dec 25;2022(1):hoab043. doi: 10.1093/hropen/hoab043 (PMC8825379; doi:10.1093/hropen/hoab043)
Supplement: hoab043_Supplementary_Data [file hoab043_supplementary_data.docx]

## **Supplementary Table SI** Example of searching strategy in the MEDLINE database.

Database: Medline (Ovid MEDLINE® Epub Ahead of Print, In-Process & Other Non-Indexed Citations, Ovid MEDLINE® Daily and Ovid MEDLINE®) 1946 to present

Papers were also retrieved from Embase (1974 to present), Web of Science Core Collection (Citation Indexes = SCI-EXPANDED, CPCI-S, CPCI-SSH, BKCI-S, ESCI) and CINAHL (via EbsscoHost) using the same searching strategy.

Search date: 12 November 2021

| 1. B-Lymphocytes/ |
| --- |
| 2. (B cell* or b-cell* or b-lymphocyte*).ti,ab,kw. |
| 3. Plasma Cells/ |
| 4. plasma cell*.ti,ab,kw. |
| 5. Antibodies/ |
| 6. antibod*.ti,ab,kw. |
| 7. Immunoglobulins/ |
| 8. immunoglobulin*.ti,ab,kw. |
| 9. Antigens, CD19/ |
| 10. CD19.ti,ab,kw. |
| 11. Antigens, CD20/ |
| 12. CD20.ti,ab,kw. |
| 13. CD22.ti,ab,kw. |
| 14. CD22.ti,ab,kw. |
| 15. Endometrium/ |
| 16. endometri*.ti,ab,kw. |
| 17. (cancer* or malign* or tumour* or tumor* or carcinoma* or adenoma* or adenocarcinoma* or neoplasm* or neoplastic* or sarcoma*).ti,ab,kw. |
| 18. 1 or 2 or 3 or 4 or 5 or 6 or 7 or 8 or 9 or 10 or 11 or 12 or 13 or 14 |
| 19. 15 or 16 |
| 20. 18 and 19 |
| 21. 20 not 17 |
| 22. limit 21 to english language |
| 23. limit 22 to human |

## **Supplementary Table SII** NEWCASTLE-OTTAWA quality assessment scores for each included study.

| First author, Year | Country/ Region | NEWCASTLE-OTTAWA Quality Score (Number of Stars) | | |
| --- | --- | --- | --- | --- |
|  |  | Selection Score | Comparability Score | Outcome Score |
| Burgener et al., 2013 | Sweden | 4 | 1 | 3 |
| Klentzeris et al., 1992 | UK | 4 | 1 | 3 |
| Chen et al., 1995 | Taiwan | 4 | 2 | 3 |
| Lucas et al., 2020 | UK | 4 | 2 | 2 |
| Fernández-Shaw et al., 1995 | UK | 4 | 2 | 3 |
| Antsiferova et al., 2005 | Russia | 4 | 2 | 3 |
| Klentzeris et al., 1995 | UK | 4 | 2 | 3 |
| Mettler et al., 1997 | Germany | 4 | 2 | 3 |
| Holzer et al., 2021 | Austria | 4 | 1 | 2 |
| Michimata et al., 2002 | Japan | 4 | 2 | 3 |
| Lachapelle et al., 1996 | Canada | 4 | 2 | 3 |
| Quenby et al., 1999 | UK | 4 | 2 | 3 |
| Bohlmann et al., 2010 | Germany | 4 | 2 | 3 |
| McQueen et al., 2021 | USA | 4 | 1 | 3 |
| Kitaya et al., 2014 | Japan | 4 | 2 | 3 |
| Li et al., 2021 | China | 4 | 2 | 2 |
| Klentzeris et al., 1994 | UK | 4 | 2 | 3 |
| Fan et al., 2021 | China | 4 | 2 | 3 |
| Zargar et al., 2020 | Iran | 4 | 2 | 2 |
| Liu et al., 2018 | China | 4 | 2 | 3 |
| Kitaya and Yasuo, 2010a | Japan | 4 | 1 | 2 |
| Toth et al., 2007 | USA | 4 | 1 | 2 |
| Cicinelli et al., 2019 | Italy | 4 | 2 | 3 |
| Song et al., 2019 | China | 4 | 1 | 2 |
| Parks et al., 2018 | USA | 4 | 1 | 3 |
| Disep et al., 2004 | UK | 4 | 2 | 2 |
| Kitaya and Yasuo, 2010b | Japan | 4 | 2 | 3 |
